# Hiding in plain sight: description of a new species of *Nyctibatrachus* (Amphibia, Anura, Nyctibatrachidae) from the central Western Ghats, India

**DOI:** 10.7717/peerj.20895

**Published:** 2026-03-27

**Authors:** C. K. Aravind, Badiger Ramesh, Chandrakanth Rukkappa Naik, K. V. Gururaja, Hebbar Priti

**Affiliations:** 1 Department of Science, Humanities and Management, Manipal Institute of Technology Bengaluru, Manipal Academy of Higher Education, Manipal, India; 2 Kali Tiger Reserve, Karnataka Forest Department, Dandeli, India; 3 Gund Wildlife Range Office, Kali Tiger Reserve, Karnataka Forest Department, Dandeli, India; 4 Dr TMA Pai Endowment Chair in Anurans, Acoustics and Anthropogenic Climate Change Lab, Srishti Manipal Institute of Art, Design and Technology, Manipal Academy of Higher Education, Manipal, India

**Keywords:** Bioacoustics, Biodiversity, Cryptic species, Endemic frogs, Freshwater ecosystem, Night frogs

## Abstract

Frogs belonging to the genus *Nyctibatrachus* are endemic to the Western Ghats biodiversity hotspot. They are the second most speciose frogs in the Western Ghats, with 70% of the species having narrow distribution ranges. They are also highly cryptic in nature. In this study, we describe a new species of *Nyctibatrachus* frog from the central Western Ghats of India. *Nyctibatrachus kali* sp. nov. is described from the Kali River basin of North Karnataka. The new species is distinguishable from all 34 currently recognised *Nyctibatrachus* species by a combination of morphological, acoustic and phylogenetic analyses. Molecular phylogeny based on two mitochondrial genes (16S rRNA and ND1) reveals that it belongs to the * N. sanctipalustris* clade. Based on the analysis of 16S rRNA, *Nyctibatrachus kali* sp. nov. shows genetic divergence >5% with its congeners, and based on the analysis of ND1, *Nyctibatrachus kali* sp. nov. shows genetic divergence >10% with its congeners. The bioacoustics analyses indicated that the new species differed from their closest congeners based on the dominant frequency of the advertisement calls and the number of notes in each call. For the first time, we observed two distinct advertisement call categories–call notes with low and high dominant frequency in *Nyctibatrachus kali* sp. nov. and its congeners. Our study adds to the rich diversity of frogs from the Western Ghats of India.

## Introduction

Tropical rainforests are known to harbour high biodiversity. They possess 62% of global terrestrial species, of which 29% are endemic, and more than 20% are prone to extinction (Pillay et al., 2022). Among vertebrates, amphibians show the highest endemism to tropical regions (33–44%), of which 39–42% are at risk of extinction (Pillay et al., 2022).

In India, the Western Ghats (WG) are a tropical biodiversity hotspot with a high diversity of amphibians (Deepak & Dinesh, 2024). Presently, 253 species are known from the Western Ghats, with 94% of species endemic to this region (Deepak & Dinesh, 2024). The family Nyctibatrachidae (Boulenger, 1882) comprises three genera: *Nyctibatrachus* and *Astrobatrachus,* endemic to the Western Ghats and *Lankanectus,* endemic to Sri Lanka. Using integrative taxonomy, many new species of *Nyctibatrachus* have been discovered in the past decade (Biju et al., 2011; Gururaja et al., 2014; Garg et al., 2017; Krutha, Dahanukar & Molur, 2017; Kumar et al., 2022). Currently, there are 34 species in this genus that are distributed across the Western Ghats (Frost Darrel, 2024). *Nyctibatrachus* frogs are nocturnal and are distinguished by rhomboid pupils and shagreened, glandular, or wrinkled skin (Biju et al., 2011). The genus not only includes large–bodied species that are adapted to torrents and fast–flowing streams (*e.g.*, *N. grandis* snout to vent length (SVL) 68 mm) but also small species (the smallest species, *N. minimus* < 11 mm) adapted to puddles, thus showing remarkable variation in their body sizes. This genus is also known to harbour cryptic species (Kumar et al., 2022); therefore, the actual species diversity in this genus could be underestimated. During one of our fieldworks for estimating the diversity of *Nyctibatrachus* species in 2021, we encountered a species that morphologically resembled *N. kumbara*. Initial assessments using molecular analysis revealed it to be distinct from *N. kumbara*.

In this study, using integrative taxonomic approaches, we conclude that the species corresponds to a distinct evolutionary lineage and describe it as a new species of *Nyctibatrachus* (Night frog) from the Kali River basin, Castlerock region of Karnataka, Central Western Ghats, India.

## Materials and Methods

### Ethics statement

Voucher specimens and tissue samples were collected following the Karnataka Forest Department research permission (PCCF(WL)/E2/CR–49/2018–19) granted to Priti Hebbar for the project Night frogs of the Western Ghats: Understanding their ecology and population genetics.

### Nomenclature and taxonomy

We follow Wiley’s (1978) evolutionary species concept, which states, “a single lineage of ancestor descendant populations of organisms which maintains its identity from other such lineages, and which has its own evolutionary tendencies and historical fate”. Using an integrative taxonomic approach by incorporating morphology, genetics and bioacoustics, we establish the identity of the new species of *Nyctibatrachus*.

Species description follows *Nyctibatrachus* taxonomical descriptions and revisions (Biju et al., 2011; Gururaja et al., 2014; Garg et al., 2017; Krutha, Dahanukar & Molur, 2017; Kumar et al., 2022). The electronic version of this article in Portable Document Format (PDF) constitutes published work according to the International Code of Zoological Nomenclature (ICZN). Therefore, the new names contained in the electronic version are considered effectively published under the Code from the electronic edition alone. This published work and the nomenclatural acts it contains have been registered with ZooBank, the official online registry of the ICZN. The ZooBank Life Science Identifiers (LSIDs) can be accessed, and the associated information viewed, through any standard web browser by appending the LSID to the prefix http://zoobank.org/. The LSID for this publication is: urn:lsid:zoobank.org:act:996A718B–844E–4C7E–A472–15718B6928D9. The online version of this work is archived and available from the following digital repositories: PeerJ, PubMed Central, SCIE, and CLOCKSS.

### Taxon sampling & specimen management

**Figure 1:**
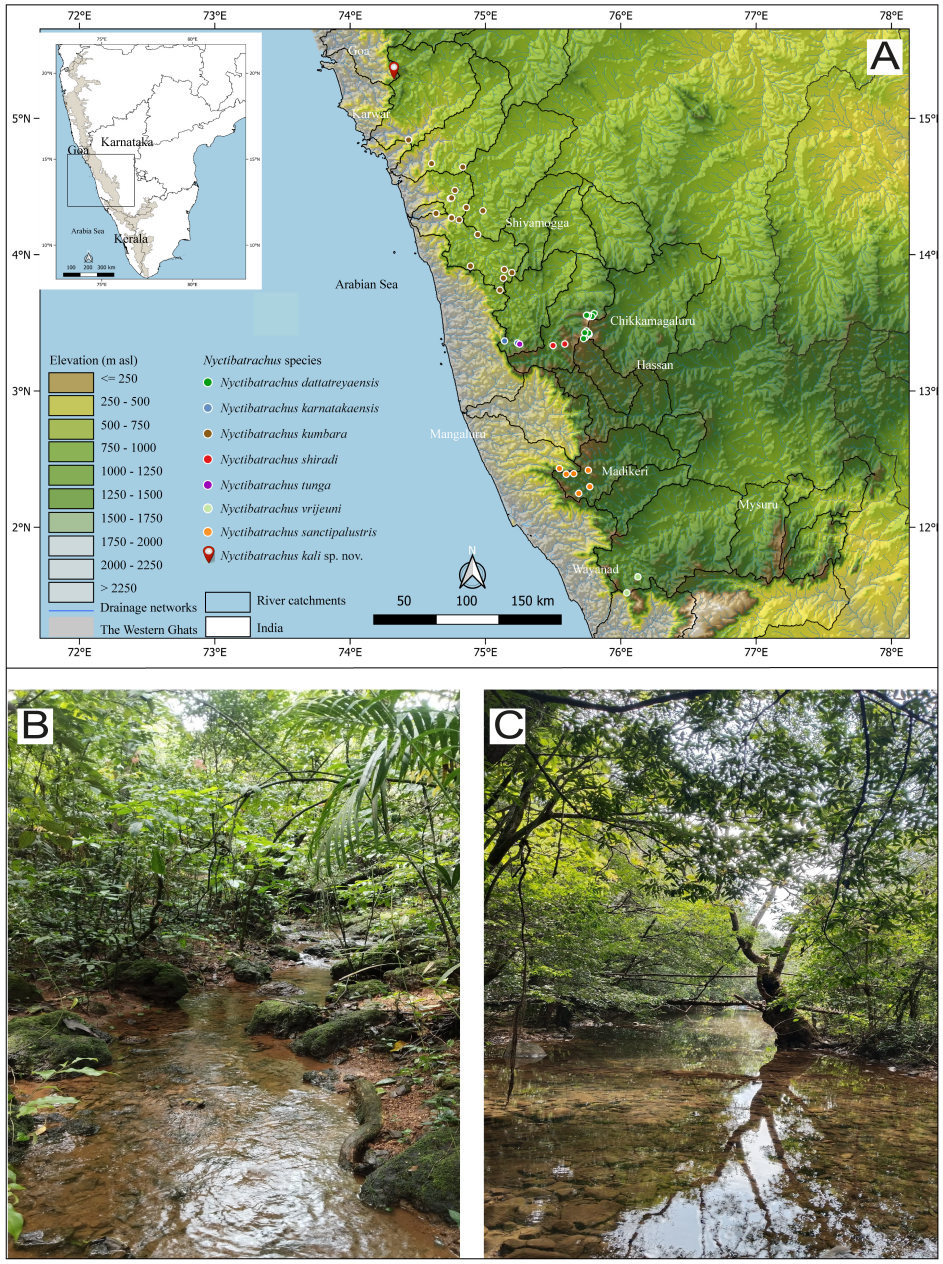
Distribution records of *Nyctibatrachus kali* sp. nov. along with its congeners from the *N. sanctipalustris* clade, Western Ghats, India. (A) Map showing the distribution of species, (B–C) Habitat of *Nyctibatrachus kali* sp.nov.

Field surveys and collection of specimens were carried out during 2021 along the streams of the evergreen and deciduous forests of the Castlerock Rock village, Dandeli Taluk, Uttara Kannada district, Karnataka state, India (15.400894°N, 74.323950°E, 578 m asl) following the Karnataka Forest Department research permission (PCCF(WL)/E2/CR–49/2018–19). The localities fall in the catchment areas of the River Kali in the Western Ghats (Fig. 1).

Adult individual frogs were located by listening to the advertisement calls and spotted using field torches. Five individuals were collected, including three adult males, one adult female, and a tadpole from Castlerock. For collecting voucher specimens, adults were euthanised, fixed in 4% formalin, and finally stored in 70% ethanol. The sample size was based on studies by Biju et al. (2011), Gururaja et al. (2014), Garg et al. (2017), and Kumar et al. (2022). The individuals’ color and natural history observations were noted at the type locality. All analyses were performed according to the norms of the Institutional Animal Ethics Committee, Manipal Academy of Higher Education (IAEC/KMC/01/2025). Voucher specimens were deposited in the Bombay Natural History Society (BNHS), Mumbai, India (Voucher numbers: BNHS 6831–6834).

### Morphological descriptions

Measurement and terminology follow Biju et al. (2011), Gururaja et al. (2014) and Kumar et al. (2022). Morphometric measurements were taken with a Mitutoyo^®^ ABS digimatic calliper (to the nearest 0.1 mm). Details of the morphometric measurements are given in Supplemental File1. The webbing formula follows Dubois, Ohler & Biju (2001). Third–finger disc and fourth–toe disc characteristics are based on Biju et al. (2011), Gururaja et al. (2014), Garg et al. (2017) and Kumar et al. (2022). Sex of the individuals was determined by examination of vocal sacs, observations of advertisement calls, the presence of nuptial pads and femoral glands in males, and the presence of eggs in females (Biju et al., 2011). The morphometric measurements from eight species used for analysis are given in Table S1 and the voucher number of the species are given in Table S2.

### Morphological comparisons

Based on descriptions of adult individuals provided by Biju et al. (2011), Gururaja et al. (2014), and Kumar et al. (2022), general morphological comparisons of known *Nyctibatrachus* species were made. To compare the collected adult individuals to those of other species known to us, we measured all three individuals of the species. Morphometric data of species that were compared with the new species were taken from Biju et al. (2011), Gururaja et al. (2014), and Kumar et al. (2022). One of us (KVG) measured the museum specimens listed below and we (ACK, KVG, and PH) verified the values by re-measuring 13 variables (SVL, head width (HW), head length (HL), snout length (SL), eye length (EL), forelimb length (FLL), hand length (HAL), maximum disc width on finger III (FDIII), maximum width of finger III (FWIII), tibia length (TL), femur length (FL), foot length (FOL), maximum disc width on toe IV (TDIV)) of the museum specimens housed at Bombay Natural History Society (BNHS), Mumbai (*N. vrijeuni* - 1 individual; *N. shiradi* - 2 individuals, *N. karnatakaensis* - 1 individual and *N. tunga* - 4 individuals); Western Ghats Regional Centre (WGRC), Zoological Survey of India (ZSI), Calicut (*N. vrijeuni* - 4 individuals; *N. shiradi* - 3 individuals, *N. karnatakaensis* - 2 individuals, *N. dattatreyaensis* - 6 individuals and *N. kumbara* - 5 individuals); and ZSI, Kolkata (*N. sanctipalustris* - 1 individual), compared it with the published data and found to be the measurements are similar to the published data.

**Figure 2:**
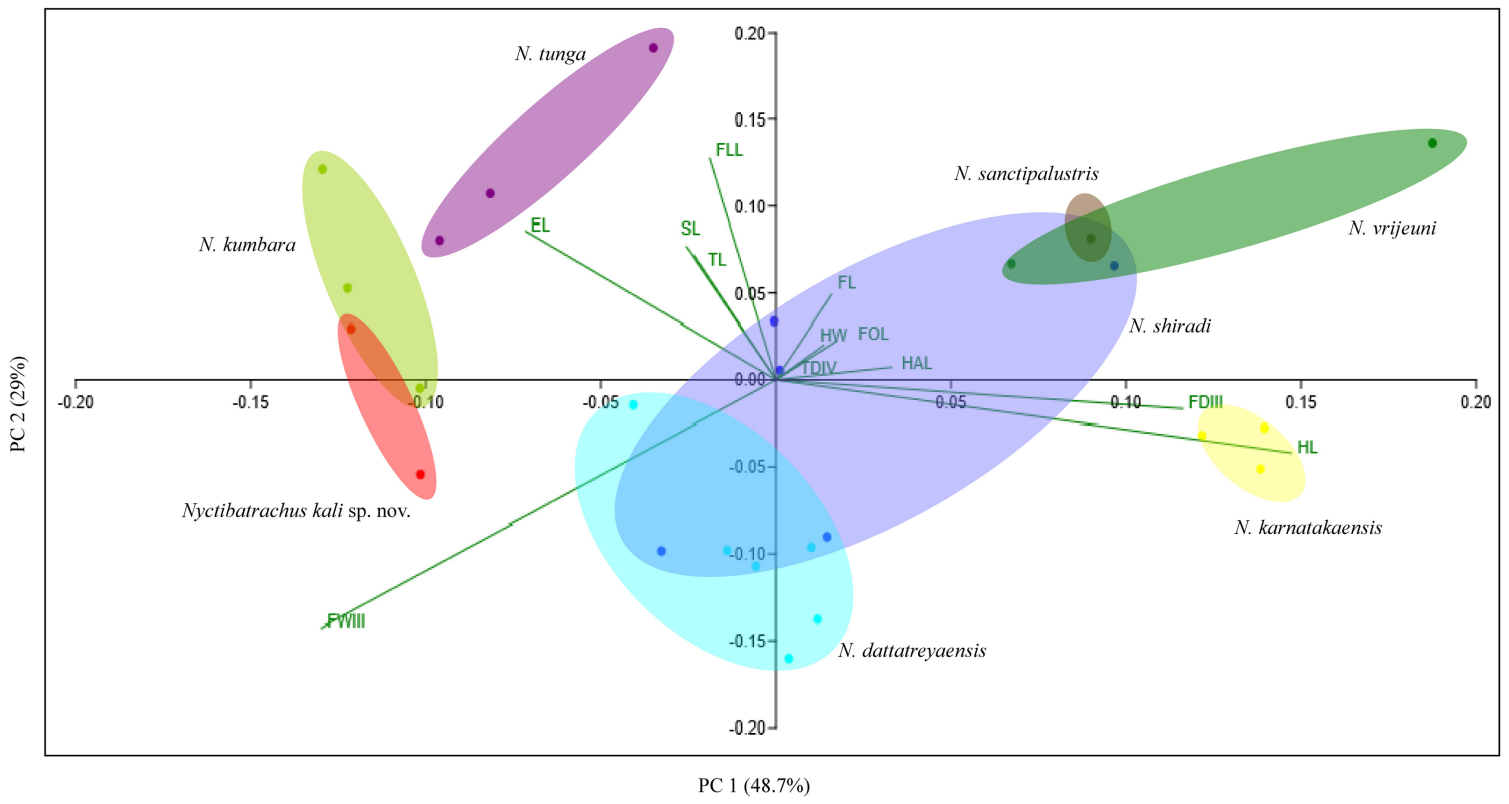
Principal Component Analysis (PCA) of eight species of the *N. sanctipalustris* clade with *Nyctibatrachus kali* sp. nov. forming distinct cluster. Component loadings are provided in Table 1.

Principal component analysis (PCA) of morphometric data from individuals of the species of the *N. sanctipalustris* clade was performed using PAST^®^ v3.2 (Hammer & Harper, 2001). Out of 61 measurements taken, only 13 were used for Principal Component Analysis (PCA) based on Kumar et al. (2022). Other measurements were used in comparisons with congeners (Biju et al., 2011). All morphometric measures were log^10^ transformed to conform to the normality requirements (Hayek, Heyer & Gascon, 2001). The measurements of 12 morphological variables (namely, HW, HL, SL, EL, FAL, HAL, FDIII, FWIII, TL, FOL, TDIV, and width of toe IV, measured at the base of disc (TWIV) of all individuals) were regressed against SVL and the effect of size was removed. For Principal Component Analysis, the residuals of each variable were considered as per Rojas et al. (2016); (Fig. 2, Table S3).

**Figure 3:**
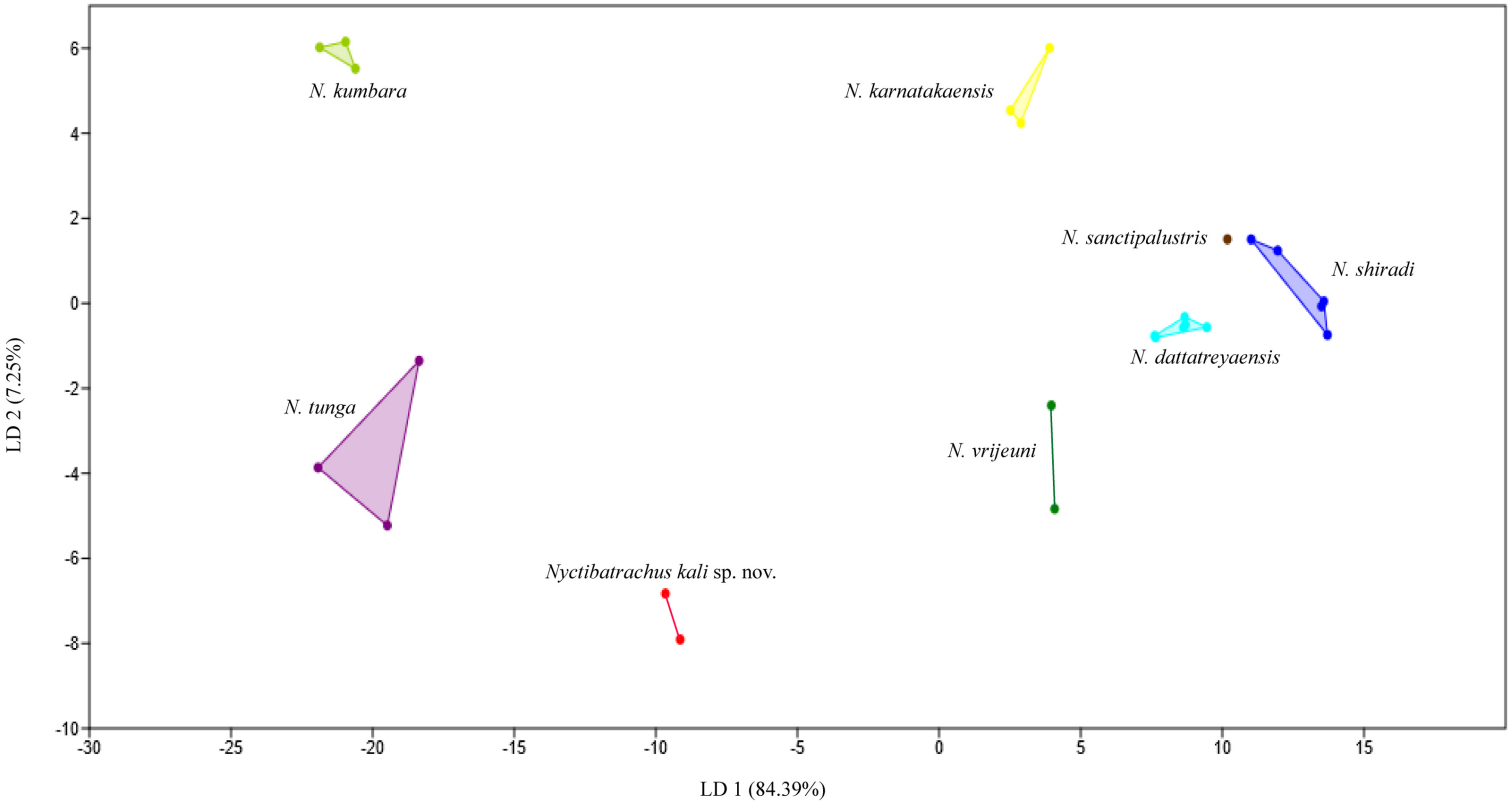
Linear Discriminant Analysis (LDA) of eight species of the *N. sanctipalustris* clade based on the ratio of SVL to HW, HL, SL, EL, FLL, HAL, FDIII, FWIII, FL, TL, FOL, and TDIV of male individuals. *Nyctibatrachus kali* sp. nov. forms a distinct cluster in the fourth quadrant of the graph. Loadings for Linear Discriminants of axes 1, 2 and 3 are given in Table 2.

Linear discriminant analysis (LDA) of morphometric data from individuals of the species of the *N. sanctipalustris* clade was performed using PAST^®^ v3.2 (Hammer & Harper, 2001) to discriminate against the two new species. Out of 61 measurements taken, only 13 were used for LDA based on Kumar et al. (2022). All morphometric measures were converted into ratio from SVL and the measurements of 12 morphological variables (namely, HW, HL, SL, EL, FAL, HAL, FDIII, FWIII, TL, FOL, TDIV, and TWIV of all individuals) were used for LDA (Fig. 3). To identify differences among species using ratios of SVL, we performed multivariate analysis of variance (MANOVA) on multivariate differences and pairwise comparisons of characters were conducted using Tukey’s Honestly Significant Difference (HSD) *post-hoc* test (Kumar et al., 2022).

### DNA extraction, amplification, and sequencing

Genomic DNA was extracted for the genetic study from three adult thigh muscle tissues and one tadpole tail tip that had been ethanol–preserved and one skin swab sample from the holotype. The extraction was performed using the salt extraction technique (Vences et al., 2012). The extracted DNA was quantified and amplified using two mitochondrial genes 16S rRNA and NADH dehydrogenase subunit 1 (ND1) using the primers 16S–F: 5′–CGCCTGTTTATCAAAAACAT–3′ and 16S–R: 5′–CCGGTCTGAACTCAGATCACGT–3′ (Palumbi et al., (1991) and NDH–L: 5′–AAACTATTTAYYAAAGARCC–3, NDH–W: 5–GGGTATGANGCTCGNATTCA–3 respectively (Roelants & Bossuyt, 2005). The reaction was performed in a 24 µl volume reaction containing one µl of template DNA (>80 ng), one µl of each primer (10 pmol/µl), 11 µl of Taq DNA Polymerase Master Mix RED and 11 µl of dH_2_O. The amplified products were sent for purification and sequencing to Barcode Biosciences Pvt. Ltd., Bengaluru, India. The GenBank Accession numbers for 16S rRNA and ND1 Genes are PV574459 –PV574460 and PV561076 –PV561077, respectively.

### Phylogenetic analyses

**Figure 4:**
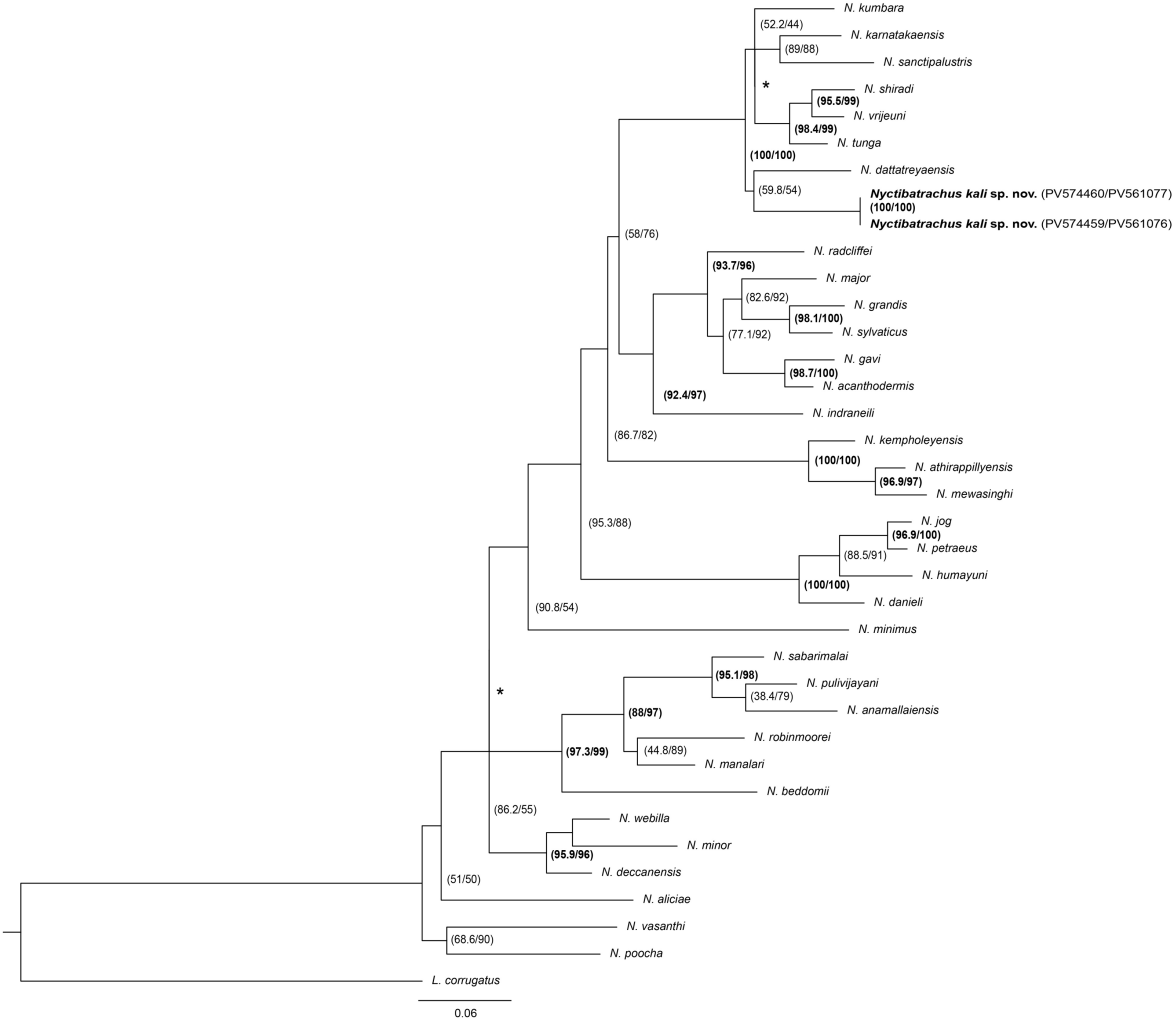
Maximum likelihood tree for *Nyctibatrachus* species and an outgroup (*Lankanectes corrugatus*) based on mitochondrial 16S rRNA and ND1 genes. Numbers in parentheses are SH-aLRT support (%)/ultrafast bootstrap support (%). * are SH-aLRT support (%)/ultrafast bootstrap support less than 50%. The Bayesian analysis support is provided as Fig. S1.

For phylogenetic analyses, sequences generated in this study were aligned with sequences of other *Nyctibatrachus* species retrieved from GenBank (Table S4). *Lankanectes corrugatus* was used as an outgroup. The sequences were assembled in MEGA v7.1 (Kumar, Stecher & Tamura, 2016) and aligned with the Muscle algorithm (Edgar, 2004). The final size of the concatenated dataset was 919 bp (16S: 468 bp and ND1: 451 bp). We performed the maximum likelihood (ML) method in IQ–TREE Web Server (Trifinopoulos et al., 2016) using a rapid bootstrap method. We inferred clade support through 1,000 replicates of an approximate likelihood ratio test with the nonparametric Shimodaira–Hasegawa correction (Anisimova et al., 2011) and ultrafast bootstrap (Hoang et al., 2018). We used MrBayes v3.2.6 (Ronquist et al., 2012) for Bayesian inference by applying the model GTR+ I+G for 16S and ND1_3rd position, TrN+I+G for ND1_1st position and TrN+G for ND1_2nd position as best–fit model suggested by the Partition finder (Lanfear et al., 2012). We used the Markov chain Monte Carlo approach, wherein two runs, each initiated from random trees, were allowed to run for 10 million generations, sampling a tree every 1,000 generations. A burn-in was set in where the first 25% of trees were discarded, and trees were constructed under a 50% majority consensus rule. The final tree was edited in FigTree v1.4.4 (Rambaut, 2018) (Fig. 4). To understand the genetic distance between species, uncorrected pairwise distances between individuals were calculated using MEGA v7.1 and are presented in Table S5 (Kumar, Stecher & Tamura, 2016).

### Bioacoustics analysis

We used active and passive acoustic recording methods for advertisement call records. Active recordings were done using a Zoom^®^ H1n handy recorder with Audio–Technica^®^ Condenser Shotgun Microphone (ATR–6550). We were unable to record the temperature and humidity at the time of sampling. However, for the remaining species, temperature, and humidity data are available (Kumar et al., 2022). Calls were recorded in .wav format at 44.1 kHz and 16 bits at a distance of ≈50 cm from the calling individuals. After the call recording, individuals were collected for SVL measurement and were released back in the same place. Passive recordings were done using an AudioMoth 1.2.0 recorder in the study sites within 2–5 m from the stream where individuals were calling. AudioMoth 1.2.0 was programmed to record 1 min every 9 min for 24 h. Recordings were made at a 48 kHz sampling rate, 16–bit, in .wav format. We manually searched for *Nyctibatrachus* species calls for further analysis in the passive acoustic records. Calls from different individuals were analysed using Raven Pro 1.6.5 (K. Lisa Yang Center for Conservation Bioacoustics at the Cornell Lab of Ornithology, 2024). In active recording data, we looked into the channels and selected the calls with a higher amplitude and frequency for the analysis. Temporal parameters are number of notes, call duration, call rise time, call fall time and the spectral parameter dominant frequency were considered for the analysis (Köhler et al., 2017).

### Other abbreviations used

PH (Priti Hebbar), ACK (Aravind C K), KVG (K V Gururaja), RB (Ramesh Badigar), CRN (Chandrakanth Rukkappa Naik), ZSIC (Zoological Survey of India, Kolkata), ZSI/WGRC/V/A (Zoological Survey of India/Western Ghats Regional Centre, Calicut/Vertebrata/Amphibia).

## Results

### Phylogenetic relationships

ML and BI analyses resulted in identical topologies; hence, only the ML tree is shown with SH–aLRT support (%)/ultrafast bootstrap support (%) (Fig. 4). The genetic analysis shows that the new species groups with clade comprising *N. sanctipalustris* (Rao, 1920), *N. karnatakaensis* Dinesh et al., 2007, *N. dattatreyaensis* Dinesh, Radhakrishnan & Bhatta, 2008, *N. shiradi* Biju et al., 2011, *N. vrijeuni* Biju et al., 2011, *N. kumbara* Gururaja et al., 2014 and *N. tunga* Kumar et al., 2022 (Fig. 4).

Genetic divergences (p–distances) for the analysed fragments of the 16S gene (468 bp) between *Nyctibatrachus kali* sp. nov. and its relatives in the *N. sanctipalustris* group, *N. kumbara*, *N. tunga*, *N. dattatreyaensis, N. karnatakaensis, N. sanctipalustris*, *N. vrijeuni* and *N. shiradi* were 5.53%, 5.53%, 5.75%, 5.75%, 6.19%, 6.19% and 6.86% respectively. Genetic divergences (p–distances) for the analysed fragments of ND1 gene between *Nyctibatrachus kali* sp. nov. and its relatives in the *N. sanctipalustris* group, *N. karnatakaensis, N. sanctipalustris*, *N. shiradi*, *N. dattatreyaensis, N. kumbara*, *N. tunga*, and *N. vrijeuni* were 10.80%, 12.44%, 12.91%, 13.38%, 13.38%, 13.62%, and 13.8% respectively. The pairwise genetic distances for all 35 nominal species of *Nyctibatrachus,* including *Nyctibatrachus kali* sp. nov., were in the range 5.53% to 13.27% (16S gene) and 10.79% to 23.94% (ND1 gene). Details are provided in Table S5.

### Morphology

Based on morphology, our study identifies a new species of *Nyctibatrachus.* The new species can be differentiated from other *Nyctibatrachus* species based on the body shape and size, fingertip and toe morphology, degree of webbing, skin colour and texture as well as dorsal folds and markings.

The principal component analysis clearly differentiates the species of the *N. sanctipalustris* clade based on their morphometry (Fig. 2). The first three components represent a total of 91.23% of the variations in the data (Table 1). A biplot of the *N. sanctipalustris* clade and 12 morphometric variables are plotted in Fig. 2. HL in PC1 (*r* = 0.598), FLL in PC2 (*r* = 0.519) and TDIV in PC3 (*r* = 0.739) are positively correlated, while FWIII in PC1 and PC2 (*r* =  − 0.527 and −0.579), and HL in PC3 (*r* =  − 0.272) are negatively correlated.

**Table 1:** Principal component analysis (PCA). Results of a PCA of 12 morphometric data (HW–Head width, HL–Head, length, SL–Snout length, EL–Eye length, FLL–Forelimb length, HAL–Hand length, FDIII–maximum disc width on finger III, FWIII–maximum width of finger III, TL–Tibia length, FL–Femur length, FOL–Foot length, and TDIV–maximum disc width on toe IV) of *N. sanctipalustris* clade consisting of eight species, viz., *Nyctibatrachus kali* sp. nov., *N. vrijeuni*, *N. sanctipalustris*, *N. shiradi*, *N. dattatreyaensis*, *N. kumbara*, *N. karnatakaensis* and *N. tunga*. All morphometric measures were log10 transformed, regressed against SVL, and the residuals of each variable were considered for PCA. PC1-PC7 refers to component loadings.

**Variables**	**PCA loadings**						
	**PC 1**	**PC 2**	**PC 3**	**PC 4**	**PC 5**	**PC 6**	**PC 7**
**HW**	0.056	0.081	−0.029	0.237	0.391	0.257	0.276
**HL**	0.598	−0.171	−0.272	0.605	−0.073	0.000	0.106
**SL**	−0.104	0.312	0.180	0.254	−0.152	0.390	0.383
**EL**	−0.290	0.348	0.159	0.191	0.578	−0.112	−0.150
**FLL**	−0.077	0.519	−0.097	−0.077	−0.400	−0.421	0.402
**HAL**	0.135	0.029	−0.053	0.024	0.323	−0.486	0.413
**FDIII**	0.472	−0.068	0.428	−0.396	0.322	−0.045	0.237
**FWIII**	−0.527	−0.579	−0.021	0.147	0.091	−0.027	0.423
**FL**	0.064	0.199	−0.247	−0.321	0.094	0.586	0.227
**TL**	−0.095	0.291	−0.190	0.294	0.166	−0.002	−0.152
**FOL**	0.071	0.092	−0.169	−0.002	0.181	−0.085	−0.307
**TDIV**	0.034	0.046	0.739	0.321	−0.197	0.050	−0.088
**Eigenvalue**	0.010	0.006	0.003	0.001	0.001	0.000	0.000
**% variance**	48.703	28.960	13.569	5.245	2.447	1.045	0.031

The results of the linear discriminant analysis of the morphometric ratio of eight species from the *N. sanctipalustris* clade are shown in Fig. 3. All eight species form distinct clusters without any overlap. Loadings for LD1, LD2 and LD3 are given in Table 2. Together, these three axes contribute to 95.47% of the total discriminants (LDA data transformation and results are in Table S6). MANOVA showed a statistically significant difference between all eight species of the *N. sanctipalustris* clade on the combined dependent variables of HW, HL, SL, EL, FLL, HAL, FDIII, FWIII, FL, TL, FOL, and TDIV, *F*_84,84_ = 3.59, *p* = 0 (Table S7).

**Table 2:** Linear discriminant analysis. Coefficients of linear discriminants of eight species of the *N. sanctipalustris* clade. Measurements of the 12 morphological variables were converted into a ratio of SVL and used in LDA. LD1-LD3 are linear discriminants. LD1, LD2 and LD3 together contribute to 95.47 the total discriminants.

**Loadings C**	**Axis 1**	**Axis 2**	**Axis 3**	**Axis 4**	**Axis 5**	**Axis 6**	**Axis 7**
**HW**	−178.93	−40.788	10.891	12.848	22.034	−56.59	−37.103
**HL**	172.09	41.493	−43.69	1.4711	15.788	15.412	6.5799
**SL**	−36.81	20.213	−70.658	−84.699	−106.82	−60.607	−9.62E+01
**EL**	−7.0554	−52.237	32.457	55.631	10.207	−31.618	103.58
**FLL**	−13.528	−4.0247	−1.6721	12.295	43.119	107.73	23.012
**HAL**	7.15E+01	18.833	59.048	2.2493	−8.167	15.208	26.244
**FDIII**	1.55E+02	260.6	468.5	−182.03	189.35	176.25	2.28E+02
**FWIII**	6.33E+02	−340.55	−2.62E+01	667.46	−89.899	332.16	153.45
**FL**	56.637	−135.55	27.592	−3.2233	−15.996	−19.372	−19.653
**TL**	−105.45	110.97	−71.716	−30.981	37.431	15.138	16.695
**FOL**	11.039	−60.327	33.54	−32.922	−22.012	−17.856	18.173
**TDIV**	−324.48	320.13	1.81E+02	−88.514	−155.88	−110.52	−2.70E+01

On pairwise comparison using Tukey’s HSD *post–hoc* test, among HW, HL, SL, EL, FLL, HAL, FDIII, FWIII, FL, TL, FOL, and TDIV variables, *Nyctibatrachus kali* sp. nov. significantly differed from *N. dattatreyaensis*, *N. karnatakaensis*, *N. sanctipalustris*, *N. shiradi* and *N. vrijeuni* in HL. Other variables did not show any significant differences between species pairs. Tukey’s HSD pair p–adjusted values are provided in the Table S8.

**Table 3:** Bioacoustics analysis. Bioacoustics analysis of seven species of *Nyctibatrachus*, including *Nyctibatrachus kali* sp. nov. The number of calls analysed in each category is given in parentheses.

**Advertisement call type**		**Dominant frequency (Hz)**	**Call duration (s)**	**Call rise time (s)**	**Call fall time (s)**
***Nyctibatrachus kali*** **sp. nov.**	**Single note call**				
AC1 (*n* = 12)	Mean ± SD	567.04 ± 57.58	0.14 ± 0.019	0.019 ± 0.005	0.121 ± 0.021
	Range	516.79–689.06	0.116–0.183	0.012–0.025	0.093–0.168
AC2 (*n* = 4)	Mean ± SD	1,981.06 ± 121.81	0.132 ± 0.047	0.023 ± 0.015	0.109 ± 0.039
	Range	1,808.79–2,067.19	0.062–0.161	0.006–0.039	0.056–0.144
***N. sanctipalustris***	**Single note call**				
AC1 (*n* = 3)	Mean ± SD	574.22 ± 49.73	0.061 ± 0.019	0.009 ± 0.003	0.053 ± 0.015
	Range	516.78–602.93	0.048–0.083	0.005–0.012	0.042–0.070
AC2 (*n* = 14)	Mean ± SD	1704.2 ± 268.53	0.129 ± 0.024	0.015 ± 0.004	0.113 ± 0.023
	Range	1,378.13–2,067.18	0.095–0.180	0.009–0.027	0.085–0.165
***N. vrijeuni***	**Single note call**				
AC1 (*n* = 9)	Mean ± SD	689.06 ± 0.0	0.038 ± 0.010	0.009 ± 0.002	0.029 ± 0.010
	Range	689.06	0.027–0.059	0.007–0.013	0.019–0.049
AC2 (*n* = 16)	Mean ± SD	2,250.22 ± 82.47	0.047 ± 0.012	0.011 ± 0.002	0.035 ± 0.012
	Range	2,067.19–2,411.72	0.034–0.080	0.006–0.014	0.023–0.069
***N. vrijeuni***	**Double note call**				
AC2 (*n* = 4)	Mean ± SD	2,239.45 ± 70.33	0.438 ± 0.029	–	–
	Range	2,153.32–2,325.59	0.403–0.473	–	–
***N. tunga***	**Single note call**				
AC1 (*n* = 5)	Mean ± SD	543.75 ± 47.39	0.076 ± 0.011	0.01 ± 0.002	0.061 ± 0.015
	Range	468.75–593.75	0.063–0.092	0.012–0.017	0.049–0.075
AC2 (*n* = 22)	Mean ± SD	1,529.83 ± 121.79	0.083 ± 0.014	0.012 ± 0.005	0.067 ± 0.016
	Range	1,312.5–1,687.5	0.058–0.108	0.009–0.025	0.046–0.092
***N. tunga***	**Double note call**				
AC2 (*n* = 2)	Mean ± SD	1,562.5 ± 88.39	0.299 ± 0.169	–	–
	Range	1,500–1,625	0.180–0.418	–	–
***N. kumbara***	**Single note call**				
AC1 (*n* = 7)	Mean ± SD	621.39 ± 23.02	0.176 ± 0.054	0.022 ± 0.004	0.154 ± 0.052
	Range	602.93–645.99	0.123–0.281	0.014–0.028	0.102–0.259
AC2 (*n* = 6)	Mean ± SD	1,658.06 ± 212.3	0.149 ± 0.042	0.023 ± 0.011	0.126 ± 0.044
	Range	1,378.13–1,894.92	0.086–0.217	0.013–0.044	0.067–0.194
***N. kumbara***	**Double note call**				
AC1 (*n* = 8)	Mean ± SD	608.31 ± 15.23	0.469 ± 0.094	–	–
	Range	602.93–645.99	0.356–0.651	–	–
AC2 (*n* = 4)	Mean ± SD	1,776.49 ± 88.78	0.422 ± 0.039	–	–
	Range	1,679.59–1,851.86	0.383–0.473	–	–
***N. shiradi***	**Single note call**				
AC2 (*n* = 14)	Mean ± SD	2,079.49 ± 88.47	0.059 ± 0.012	0.014 ± 0.005	0.044 ± 0.011
	Range	1,894.92–2,239.45	0.033–0.074	0.006–0.024	0.016–0.059
***N. shiradi***	**Double note call**				
AC2 (*n* = 13)	Mean ± SD	2,085.64 ± 96.62	0.387 ± 0.035	–	–
	Range	1,981.06–2,239.45	0.351–0.457	–	–
***N. shiradi***	**3 Note call**				
AC2 (*n* = 2)	Mean ± SD	1,937.99 ± 60.91	0.779 ± 0.132	–	–
	Range	1,894.92–1,981.06	0.685–0.872	–	–
***N. dattatreyaensis***	**Single note call**				
AC1 (*n* = 9)	Mean ± SD	622.07 ± 37.98	0.065 ± 0.012	0.007 ± 0.002	0.059 ± 0.012
	Range	602.93–689.06	0.071–0.082	0.008–0.008	0.062–0.075
AC2 (n24)	Mean ± SD	2,436.84 ± 112.1	0.078 ± 0.009	0.007 ± 0.002	0.071 ± 0.009
	Range	2,325.59–2,670.12	0.058–0.078	0.005–0.008	0.053–0.071
***N. dattatreyaensis***	**Double note call**				
AC2 (*n* = 6)	Mean ± SD	2,454.79 ± 105.49	0.404 ± 0.026	–	–
	Range	2,325.59–2,583.98	0.386–0.455	–	–
***N. dattatreyaensis***	**4 Note call**				
AC2 (*N* = 4)	Mean ± SD	2,713.18 ± 149.19	1.029 ± 0.021	–	–
	Range	2,497.85–2,842.38	0.998–1.045	–	–
***N. dattatreyaensis***	**5 Note call**				
AC2 (*n* = 3)	Mean ± SD	2,383.01 ± 348.1	1.336 ± 0.031	–	–
	Range	2,067.19–2,756.25	1.307–1.368	–	–

### Bioacoustics analysis

**Figure 5:**
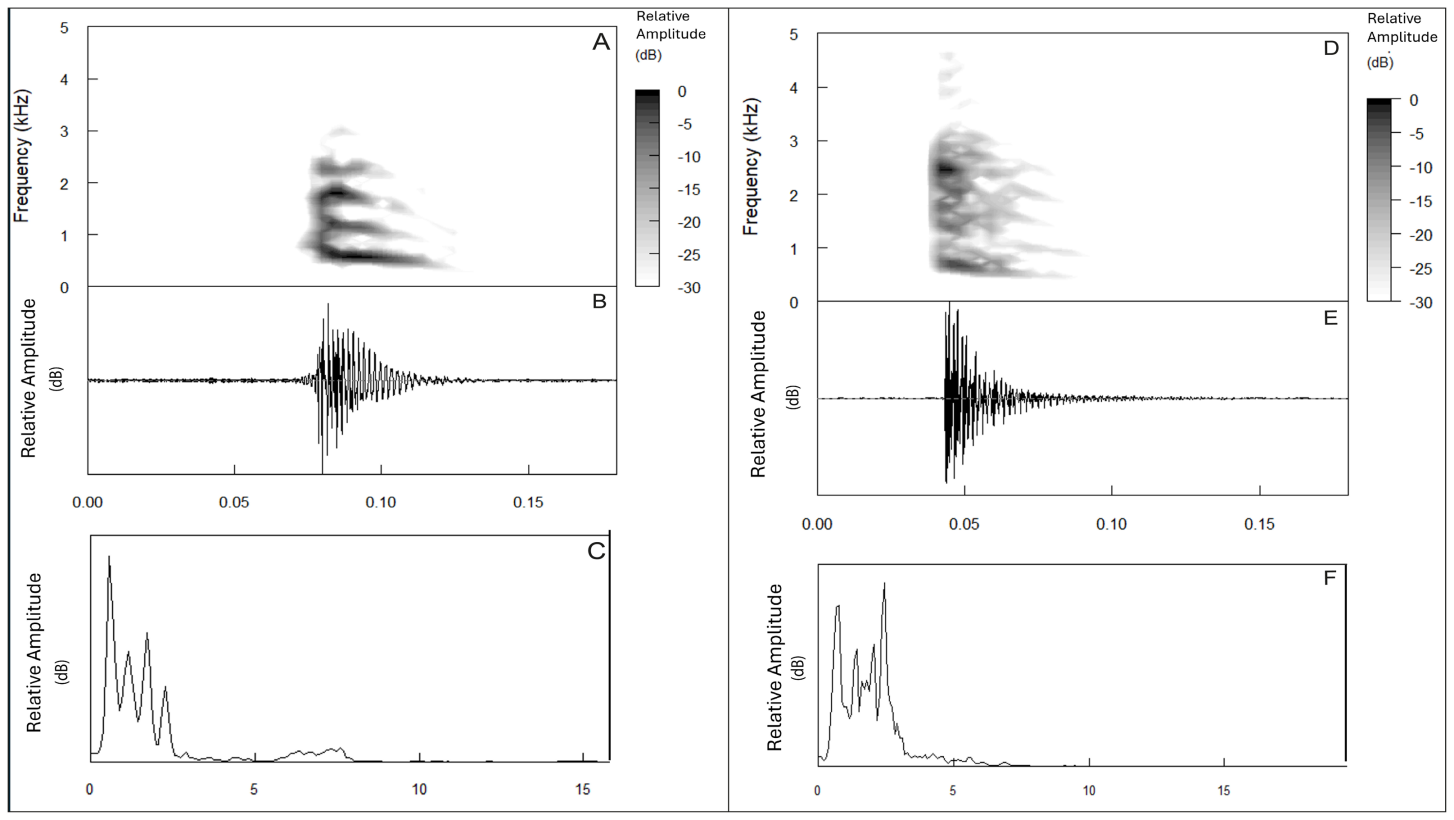
Advertisement call of *Nyctibatrachus kali* sp. nov. (A) Oscillogram of AC1, (B) Spectrogram of AC1, (C) Power spectrum of AC1, (D) Oscillogram of AC2, (E) Spectrogram of AC2, and (F) Power spectrum of AC2.

Advertisement calls were observed and recorded only in males, as females did not vocalise. Calls (*n* = 16) consisted of a single note (tok) with two distinct dominant frequencies. Twelve calls had a lower dominant frequency (advertisement call 1; AC1), and four calls had a higher dominant frequency (advertisement call 2; AC2). The calling activity of the individuals was observed from 18:00 h to 21:00 h. The call characteristics of *Nyctibatrachus kali* sp. nov. are given in Table 3 and Fig. 5. The dominant frequency of AC1 was 567.04 ± 57.58 Hz (range 516.80–689.06 Hz) with call duration of 0.140 ± 0.019 s. Dominant frequency of AC2 was 1,981.06 ± 121.81 Hz (range 1,808.79–2,067.19 Hz) with call duration 0.132 ± 0.047 s. Details of call rise time and fall time for single note calls are given in Table 3. Three to four harmonics have been observed in the calls of new species and other species of *Nyctibatrachus* whose calls were analysed here.

**Figure 6:**
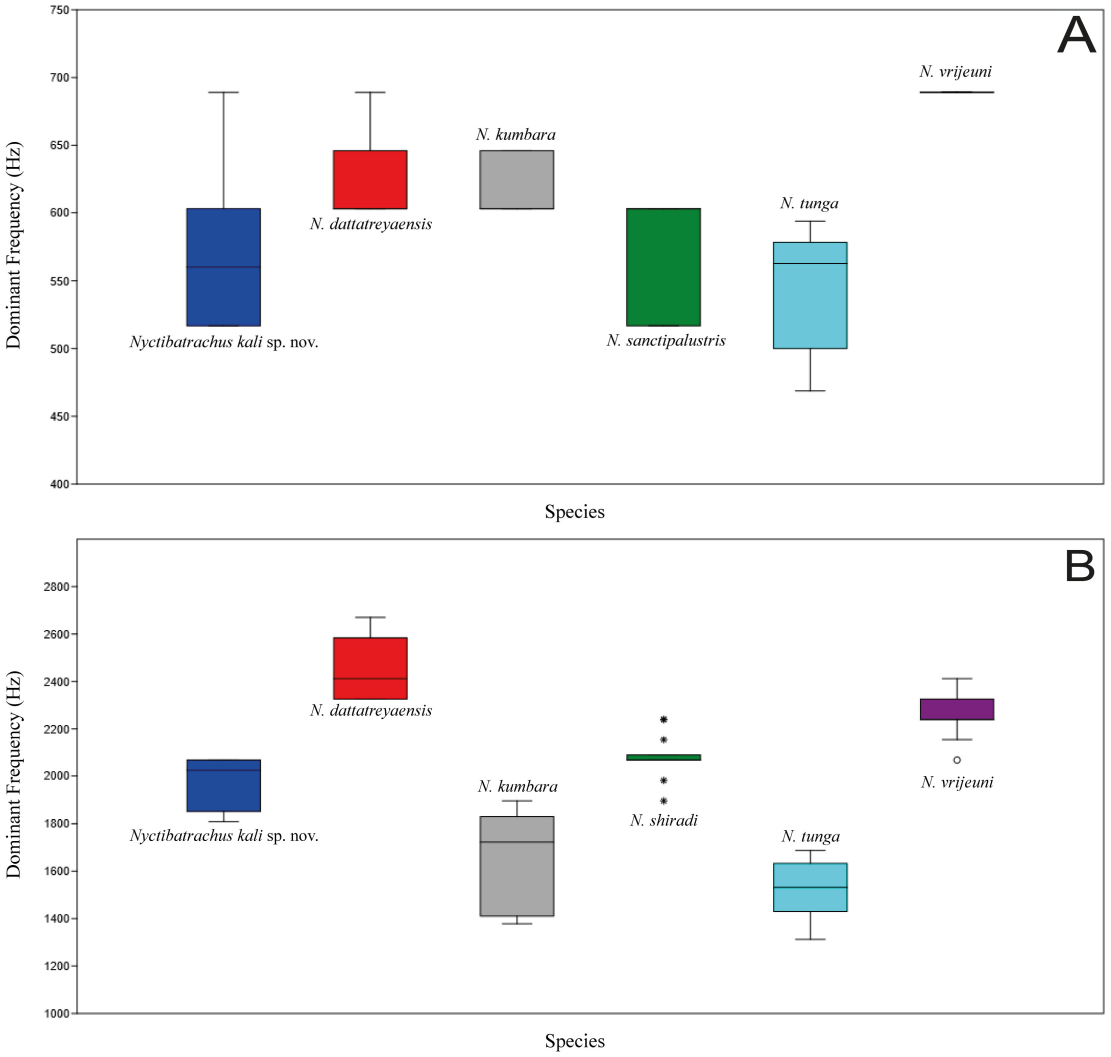
Box plot of dominant frequency (Hz) of advertisement call in *N. sanctipalustris* clade, including *Nyctibatrachus kali* sp. nov. (A) Advertisement call 1 (AC1) and (B) advertisement call 2 (AC2). Temperature and relative humidity information are not available. Mann–Whitney U test details are provided in Table S7.

Mann–Whitney U tests were performed to understand the significance of the difference between dominant frequencies of all species in the *N. sanctipalustris* clade except *N. karnatakaensis*, in which AC1 of *Nyctibatrachus kali* sp. nov. shows significant differences between *N. dattatreyaensis* (*p* = 0.025), *N. kumbara* (*p* = 0.026) and *N. vrijeuni* (*p* = 0.001) in terms of the dominant frequency. AC2 of *Nyctibatrachus kali* sp. nov. is significantly different from AC2 of *N. dattatreyaensis* (*p* = 0.003), *N. kumbara* (*p* = 0.032), *N. sanctipalustris* (*p* = 0.060, marginally significant), *N. tunga* (0.002), and *N. vrijeuni* (*p* = 0.003) in terms of the dominant frequency (Figs. 5 and 6, Table S9). Details of other call parameters are shown in Table 3.

AC1 (low frequency) of *Nyctibatrachus kali* sp. nov. shows significant differences between *N. dattatreyaensis*, *N. kumbara* and *N. vrijeuni* in terms of the dominant frequency, whereas *Nyctibatrachus kali* sp. nov. shows no significant differences from *N. sanctipalustris*, *N. tunga*, and *N. shiradi* based on AC1 dominant frequency.

AC2 (high frequency) of *Nyctibatrachus kali* sp. nov. is significantly different from all the other species except *N. shiradi* in terms of the dominant frequency. So far from the call recordings available (which are recorded by us), the differences in dominant frequency are significant.

*Nyctibatrachus kali* sp. nov. can be differentiated from *N. tunga*, and *N. shiradi* based on the number of notes present in the call. *Nyctibatrachus kali* sp. nov. is observed to produce single-note calls, whereas *N. tunga* has single as well as double-note calls, and *N. shiradi* has calls up to three notes. *Nyctibatrachus kali* sp. nov. cannot be differentiated from *N. sanctipalustris* in advertisement call, where both species have same number of notes (single note) in their call.

In conclusion, our findings from molecular genetics, morphology, and bioacoustics analyses offer independent evidence supporting a distinct lineage that has not yet been described and is hereby designated as a new species of *Nyctibatrachus* from the Western Ghats.

## Systematics

### Generic placement

This species is assignable to the genus *Nyctibatrachus* because of its medium to large size, rhomboidal–shaped pupil, glandular wrinkled skin, presence of vomerine teeth, notched tongue, finger and toes with discs, absence of webbing on fingers and presence of webbing on toes, presence of subocular gland, and semi–aquatic to aquatic habitat (Biju et al., 2011, Kumar et al., 2022).

### Species accounts

**Table:** 

New species
*Nyctibatrachus kali* sp. nov.
LSID urn:lsid:zoobank.org:act:996A718B–844E–4C7E–A472–15718B6928D9
Proposed standard English name. Kali Night Frog

### Holotype

BNHS 6831, adult male, from Castlerock (15.400894°N, 74.323950°E, 578 m asl), Dandeli Taluk, Uttara Kannada District, Karnataka state, India, collected by PH, GKV, RB and CRN on 4th November 2021.

### Paratypes

BNHS 6832, one adult male, and BNHS 6833, one adult female, were collected from the same locality as the holotype by the team on 4th November 2021.

### Referred material

BNHS 6834, one sub-adult, partially deformed and not used in morphometric and molecular analysis.

### Etymology

We name the species as Kali, the river at which the type locality of the species located. The specific epithet is an invariable noun in apposition.

### Diagnosis

Based on molecular phylogenetic analysis, *Nyctibatrachus kali* sp. nov. belongs to the *N. sanctipalustris* clade (Fig. 4) and is a sister lineage to *N. dattatreyaensis*. Hence, we compared *Nyctibatrachus kali* sp. nov. (both ♂ and ♀ individuals of type specimens) with all the species belonging to the *N. sanctipalustris* clade (both ♂ and ♀ individuals of type specimens), *i.e., N. dattatreyaensis*; *N. karnatakaensis*; *N. kumbara*; *N. sanctipalustris*; *N. shiradi*, *N. tunga* and *N. vrijeuni*.

*Nyctibatrachus kali* sp. nov. can be distinguished from known congeners by the following combination of characters: (1) medium male adult size (SVL 46.7–48.1 mm, *N* = 2); (2) head width is 1.3–1.4 times larger than head length (male HW/HL ratio 1.34 ± 0.03); (3) a well–developed ridge extending from the lip over the tip of the snout to between the nostrils, at which point it bifurcates, producing an inverted ‘Y’; (4) third finger disc slightly wider than finger width (male FD_III_ 0.8–1.0, FW_III_ 0.7–0.9), without dorso–terminal groove; (5) fourth toe disc moderately wider than toe width (male TD_IV_ 1.3–1.5, TW_IV_ 0.6–0.9), with dorso–terminal groove and cover notched distally; (6) presence of two palmar tubercles; (7) foot webbing moderately large, fourth toe webbing nearly extending up to the third subarticular tubercle on one side and below the toe disc on the other side of toe IV (I 0–1 II 0–2 III 0–0 IV 3–0 V) nearly extending up to the first subarticular tubercle on either side; (8) femur slightly longer than tibia (male FL/TL ratio 1.08 ± 0.04%); (9) tibia nearly equal to foot length (male TL/FOL ratio 1.02 ± 0.09%); (10) single–note calls, two types of advertisement call (AC), the dominant frequency of AC1 and AC2 were 567.04 ± 57.58 Hz (range 516.80–689.06 Hz) and 981.06 ± 121.81 Hz (range 1,808.79–2,067.19 Hz) respectively.

## Comparisons with other species

*Nyctibatrachus kali* sp. nov. is morphologically different to *N. dattatreyaensis* in size (SVL 46.7–48.1 mm ♂, 43.3 mm ♀ *vs* 36.2–42.3 mm ♂, 38.8 mm ♀). Head wider than long in *N. kali* sp. nov. (HW 18.5–19.5 mm ♂, 16.5 mm ♀, HL 14.0–14.4 mm ♂, 12.6 mm ♀) *vs.* head equal to sub–equal than long (HW 15–18.4 mm ♂, 15.5 mm ♀, HL 15–18.4 mm ♂, 15.3–15.4 mm ♀). Ratios of HW/HL were consistent in all individual specimens deposited in the museum and in males and female (♂, 1.34 ± 0.03 and ♀, 1.31 and *N. dattatreyaensis*, ♂, 1 ± 0.003 and ♀, 1 ± 0.005). *N. kali* sp. nov. can be further distinguished from *N. dattatreyaensis*, based on acoustic parameters: Advertisement call type 1 and 2 of *N. kali* sp. nov. shows significant differences between *N. dattatreyaensis* based on dominant frequency, as well as based on the number of notes present in the calls, where the calls of *Nyctibatrachus kali* sp. nov. show single–note calls, whereas *N. dattatreyaensis* shows multiple notes in the call that go up to five notes.

*Nyctibatrachus kali* sp. nov. is very distinct and smaller in size compared to *N. karnatakaensis* (*N. kali* sp. nov. SVL 46.7–48.1 mm ♂, 43.3 mm ♀ *vs N. karnatakaensis*, SVL 56–61.9 mm ♂, 63.8 mm ♀). Tips of fingers with a weakly developed disc (FDIII 0.8–1 mm ♂, 1.9 mm ♀), third finger disc without dorso–terminal groove, and tips of toes with a moderately developed disc (TDIV 1.3–1.5 mm ♂, 1.7 mm ♀) and fourth toe disc with dorso–terminal groove, cover notched distally in *N. kali* sp. nov. *vs.* finger and toe discs well developed (FDIII 1.5–1.6 mm ♂, 1.5 mm ♀, TDIV 2–2.2 mm ♂, 2.8 mm ♀) with dorso–terminal groove, cover rounded distally in *N. karnatakaensis*. Webbing medium, reaching the third subarticular tubercle on either side of toe IV *Nyctibatrachus kali* sp. nov. *vs* webbing extensive, reaching well beyond the third subarticular tubercle on either side of toe IV in *N. karnatakaensis.*

*Nyctibatrachus kali* sp. nov. is similar to *N. kumbara* in size (*N. kali* sp. nov. SVL 46.7–48.1 mm ♂, 43.3 mm ♀ *vs N. kumbara*, SVL 46–47.4 mm ♂, 42.8–43.3 mm ♀). Forelimbs (FLL 9.6–10.2 mm ♂, 8.1 mm ♀) strong and smaller than hand length (HAL 11.4–11.6 mm ♂, 12.4 mm ♀) in *N. kali* sp. nov. *vs* forelimbs (FLL 9.6–10.9 mm ♂, 8.1–9.1 mm ♀) strong and sub–equal to hand length (HAL 10.1–12.1 mm ♂, 10.4–11 mm ♀) in *N. kumbara*. Third finger disc without dorso–terminal groove and fourth toe disc with dorso–terminal groove, cover notched distally in *Nyctibatrachus kali* sp. nov. *vs.* third finger discs with dorso–terminal groove, cover notched distally, and fourth toe disc with dorso–terminal groove, cover bifurcate distally in *N. kumbara*.

*Nyctibatrachus kali* sp. nov. is very distinct from *N. sanctipalustris* in size (*N. kali* sp. nov. SVL 46.7–48.1 mm ♂, 43.3 mm ♀ *vs N. sanctipalustris*, SVL 33.8 mm ♂, 25.7–37.6 mm ♀). Head wider than long in *Nyctibatrachus kali sp. nov.* (HW 18.5–19.5 mm♂, 16.5 mm ♀, HL 14.0–14.4 mm ♂, 12.6 mm ♀) *vs.* head equal to sub–equal than long in *N. sanctipalustris* (HW 14.1 mm ♂, 10.7 mm ♀, HL 15.5 mm ♂, 9.2 mm ♀). Webbing medium in *Nyctibatrachus kali* sp. nov., (in all type specimen) reaching third subarticular tubercle on one side and below the toe disc on the other side of toe IV (I 0–1 II 0–2 III 0–0 IV 3–0 V) *vs. N. sanctipalustris* (in lectotype; Biju et al., 2011) webbing medium, reaching the third subarticular tubercle on either side of toe IV (I 0–$ \frac{1}{2} $ II 0–2 III 0–3IV 3–0 V).

**Figure 7:**
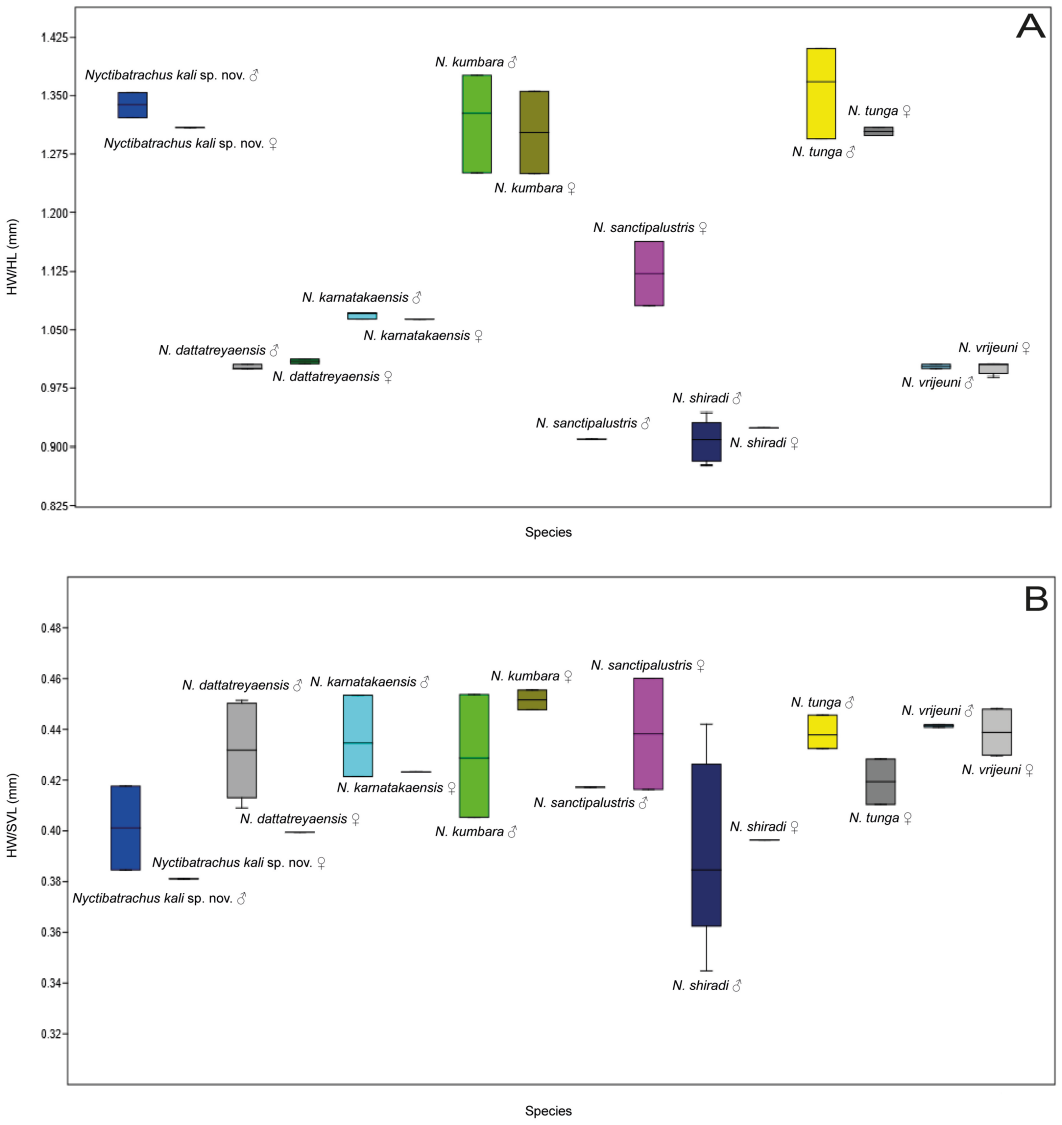
HW/HL and HW/SVL ratio in individuals. (A) Box plot of HW/HL ratio in male and female individuals of eight species of the *N. sanctipalustris* clade including *Nyctibatrachus kali* sp. nov. (B) Box plot of HW/SVL in male and female individuals of eight species of the *N. sanctipalustris* clade including *Nyctibatrachus kali* sp. nov.

*Nyctibatrachus kali* sp. nov. is very distinct from *N. shiradi* in size (*Nyctibatrachus kali* sp. nov. SVL 46.7–48.1 mm ♂, 43.3 mm ♀ *vs N. shiradi* SVL 18.1–22.1 mm ♂, 27.5 mm ♀). Head wider than long in *Nyctibatrachus kali* sp. nov. (HW 18.5–19.5 mm ♂, 16.5 mm ♀, HL 14.0–14.4 mm ♂, 12.6 mm ♀) *vs.* head width sub–equal to length in *N. shiradi* (HW 7.0–8.5 mm ♂, 10.9 mm ♀, HL 7.9–9.7 mm ♂, 11.8 mm ♀). Ratio of HW/HL were consistent in all individual specimens deposited in the museum as well as in male and female individuals (*Nyctibatrachus kali* sp. nov, ♂, *n* = 2, 1.34 ±0.03 and ♀, *n* = 1, 1.31 and *N. shiradi*, ♂, *n* = 5, 0.9 ± 0.03 and ♀, *n* = 1, 0.9). Figure 7 represent the HW/HL and HW/SVL ratios in these two species. In all the specimens of *Nyctibatrachus kali* sp. nov. fourth toe disc with dorso–terminal groove, cover notched distally *vs.* third finger discs with dorso–terminal groove, cover notched distally, and fourth toe disc with dorso–terminal groove, cover notched distally in *N. shiradi* (Holotype, ZSI/WGRC/V/A/815). Webbing medium in *Nyctibatrachus kali* sp. nov., (in all type specimen) reaching third subarticular tubercle on one side and below the toe disc on the other side of toe IV (I 0–1 II 0–2 III 0–0 IV 3–0 V) *vs. N. shiradi* (in Holotype) webbing small, barely reaching beyond second subarticular tubercle on either side of toe IV (I 0–$ \frac{1}{4} $ II 0–2 III 0–2 $ \frac{1}{2} $ IV 2$ \frac{1}{2} $–0 V).

*Nyctibatrachus kali* sp. nov. is similar to *N. tunga* in size (*Nyctibatrachus kali* sp. nov. SVL 46.7–48.1 mm ♂, 43.3 mm ♀ *vs. N. tunga*, SVL 37–40.2 mm ♂, 42.4–47.4 mm ♀). Head wider than long in *Nyctibatrachus kali sp. nov.* (HW 18.5–19.5 mm ♂, 16.5 mm ♀, HL 14.0–14.4 mm ♂, 12.6 mm ♀) *vs.* head equal to sub–equal than long in *N. tunga* (HW 17.2–18 mm ♂, 17–20 mm ♀, HL 11.7–13.6 mm ♂, 13.4–15.5 mm ♀). Webbing medium in *Nyctibatrachus kali* sp. nov. (in all type specimens), reaching third subarticular tubercle on one side and below the toe disc on the other side of toe IV (I 0–1 II 0–2 III 0–0 IV 3–0 V) *vs. N. tunga* (in holotype) webbing medium, reaching third subarticular tubercle on either side of toe IV (I 0–$ \frac{1}{2} $ II 0–2 III 0–3 IV 3–0 V).

*Nyctibatrachus kali* sp. nov. is different to *N. vrijeuni* in size (*Nyctibatrachus kali* sp. nov. SVL 46.7–48.1 mm ♂, 43.3 mm ♀ *vs N. vrijeuni* SVL 38.7–43.1 mm ♂, 37.4–42.5 mm ♀). Head wider than long in *Nyctibatrachus kali* sp. nov. (HW 18.5–19.5 mm ♂, 16.5 mm ♀, HL 14.0–14.4 mm ♂, 12.6 mm ♀) *vs.* head equal to sub–equal than long in *N. vrijeuni* (HW 17.1–19 mm ♂, 16.1–19.0 mm ♀, HL 17.0–19.0 mm ♂, 16.0–18.9 mm ♀). Ratios of HW/HL were consistent in all individual specimens deposited in the museum and in male and female (*Nyctibatrachus kali* sp. nov., ♂, 1.34 ± 0.03 and ♀, 1.31; and *N. vrijeuni*, ♂, 1.0 ± 0.0 and ♀, 1.0 ± 0.01). In all the specimens of *Nyctibatrachus kali* sp. nov. fourth toe disc with dorso–terminal groove, cover notched distally *vs.* fourth toe disc with dorso–terminal groove, cover rounded distally in *N. vrijeuni* (holotype). Webbing medium in *Nyctibatrachus kali* sp. nov. (in all type specimens), reaching third subarticular tubercle on one side and below the toe disc on the other side of toe IV (I 0–1 II 0–2 III 0–0 IV 3–0 V) *vs. N. vrijeuni* (in holotype) webbing medium, barely reaching the third subarticular tubercle on either side of toe IV (I 0–1 II 0–2 III 0–$2 \frac{3}{4} $ IV 2$ \frac{3}{4} $–0 V).

### Description of the holotype

**Figure 8:**
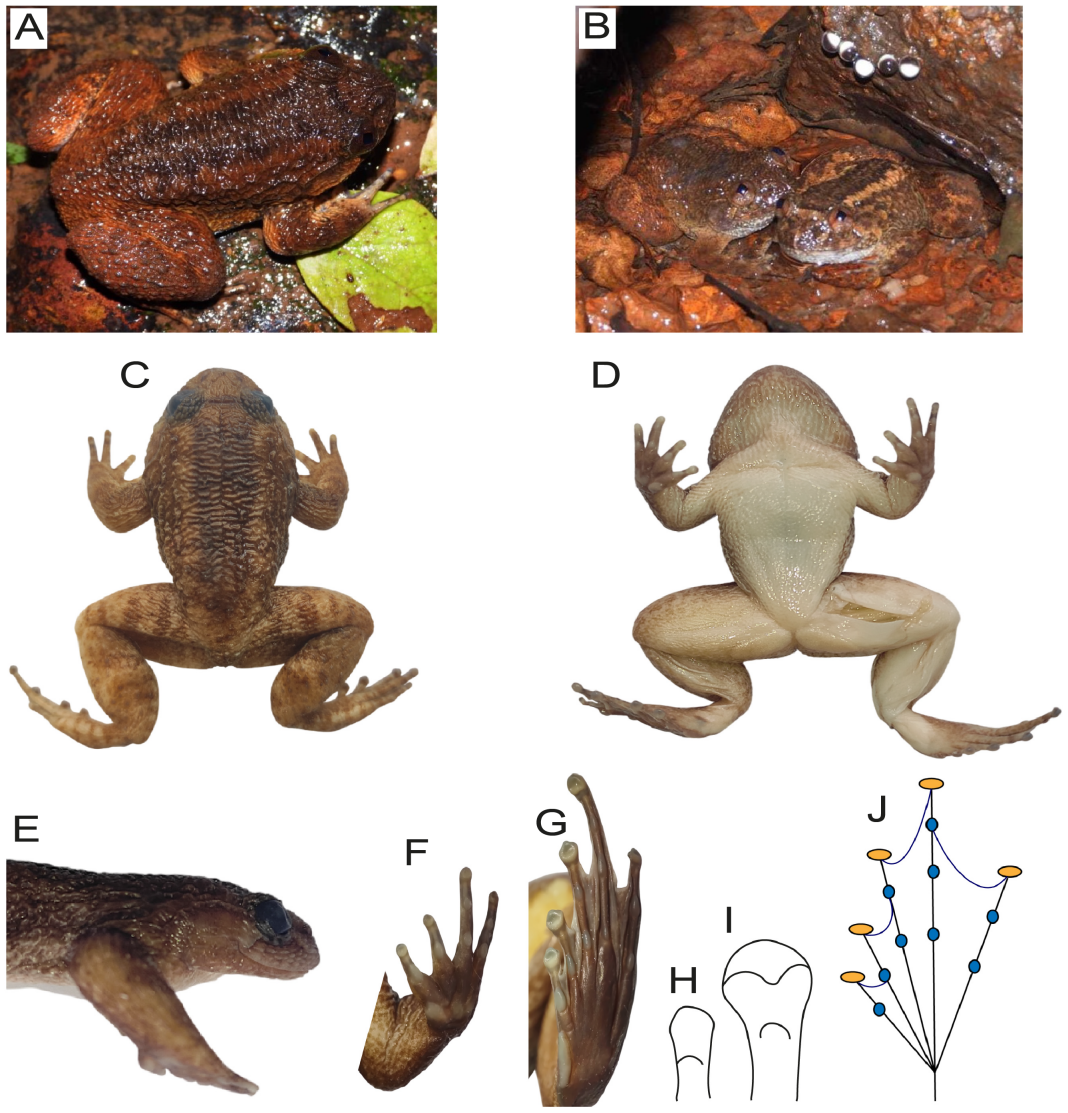
Habitus of holotype (BNHS 6831) of *Nyctibatrachus kali* sp. nov. (A) Live individual of holotype; (B) Slow flowing stream habitat of the holotype with female and a clutch of eggs; (C) Dorsal view; (D) Ventral view; (E): Lateral profile of head and trunk; (F): Ventral view of hand; (G): Ventral view of foot; (H): third finger disc without dorso-terminal groove; (I): fourth toe disc with dorso-terminal groove cover notched distally; (J): Schematic view of webbing in hindlimb (black lines represents toes, curved black lines represents webbing, blue circles represents subarticular tubercle and orange circles represents toe discs.).

BNHS 6831, adult male (Figs. 8A, 8J), *Morphometric* data are given in Table 4. A medium–sized species of *Nyctibatrachus* (SVL 46.7 mm); habitus compact and squat; head wider (HW 19.5 mm) than long (HL 14.4 mm; distance from the rear of the mandible to the center of the nostril (MN) 11.7 mm; distance from the rear of the mandible to the anterior–most orbital border (MFE) 8.6 mm; distance from the rear of the mandible to the posterior–most orbital border (MBE) 3.4 mm), rounded in frontal view; snout rounded in dorsal view, marginally protruding, its length longer (SL 6.8 mm) than horizontal diameter of eye (EL 4.8 mm); canthus rostralis rounded in dorsal view; loreal region concave; interorbital space flat (IUE 5 mm) and twice upper eyelid width (UEW 2.3 mm); much greater than internarial distance (IN 4.2 mm); distance between back of eyes (IBE 15.2 mm) almost twice distance between front of eyes (IFE 8.1 mm); nostrils oval, without flap of skin, closer to eye (EN 3.0 mm) than to snout tip (NS 4.2 mm); eye large (EL/HL = 0.34) protruding on sides of head, its diameter greater than eye to nostril distance (EL/EN = 1.62); pupil rhomboidal; tympanum indistinct; subocular gland distinct (Fig. 8E); pineal ocellus absent; vomerine ridge oval, posterior to choanae, slightly oblique, bearing six spinose teeth on left and five on right ridge; symphysial knob ‘ 
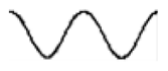
’ shaped, moderately developed; tongue moderate, oval emarginate, median lingual process absent; parotoid glands, cephalic ridges and co–ossified skin absent.

**Table 4:** Morphometric measurements (in mm) of the type series of *Nyctibatrachus kali* sp. nov.

**Species**	***Nyctibatrachus kali*** ** sp. nov.**				
**Type**	**Holotype** **BNHS 6831**	**Paratype** **BNHS 6832**	**Mean ± SD**	**Range**	**Paratype** **BNHS 6833**
**Sex**	**Male**	**Male**			**Female**
SVL	46.7	48.1	47.39 ± 0.957	46.7–48.1	43.3
EL	4.8	5.6	5.19 ± 0.528	4.8–5.6	4.8
EN	3.0	2.7	2.84 ± 0.184	2.71–2.97	4.2
HL	14.4	14.0	14.21 ± 0.231	14.0–14.4	12.6
HW	19.5	18.5	19 ± 0.731	18.5–19.5	16.5
HD	8.2	8.4	8.34 ± 0.137	8.2–8.4	6.4
IBE	15.2	15.5	15.36 ± 0.212	15.2–15.5	14.6
IFE	8.1	8.2	8.18 ± 0.054	8.1–8.2	7.4
IN	4.2	3.9	4.03 ± 0.21	3.9–4.2	3.9
IUE	5.0	5.2	5.12 ± 0.17	5.0–5.2	5.1
MBE	3.4	3.8	3.64 ± 0.28	3.4–3.8	2.9
MFE	8.6	9.1	8.82 ± 0.361	8.6–9.1	7.3
MN	11.7	11.7	11.68 ± 0.028	11.7–11.7	10.3
NS	4.2	3.4	3.8 ± 0.533	3.4–4.2	3.2
SL	6.8	6.7	6.77 ± 0.061	6.7–6.8	7.1
UEW	2.6	2.8	2.7 ± 0.144	2.8–2.6	2.7
fd1	1.1	0.8	0.952 ± 0.214	0.8–1.1	1.4
fd2	1.1	0.8	0.96 ± 0.212	0.8–1.1	1.6
fd3	1.0	0.8	0.887 ± 0.193	0.8–1.0	1.9
fd4	1.0	0.8	0.915 ± 0.12	0.8–1.0	1.6
fw1	0.8	0.7	0.7 ± 0.071	0.7–0.8	0.7
fw2	0.8	0.7	0.74 ± 0.094	0.7–0.8	0.8
fw3	0.9	0.7	0.785 ± 0.181	0.7–0.9	0.7
fw4	0.8	0.7	0.747 ± 0.094	0.7–0.8	0.6
FFL	3.7	3.0	3.37 ± 0.481	3.0–3.7	3.7
SFL	3.9	3.4	3.68 ± 0.332	3.4–3.9	4.3
TFL	4.8	5.3	5.06 ± 0.325	4.8–5.3	6.0
FrFL	3.7	3.8	3.78 ± 0.082	3.8–3.7	5.5
HAL	11.4	11.6	11.51 ± 0.12	11.4–11.6	12.4
FLL	9.6	10.2	9.92 ± 0.401	9.6–10.2	8.1
AG	12.7	13.5	13.11 ± 0.549	12.7–13.5	12.4
BW	18.9	21.4	20.16 ± 1.74	18.9–21.4	14.8
BWG	12.4	19.6	15.97 ± 5.07	12.4–19.6	11.2
FGL	9.2	8.7	8.97 ± 0.349	8.7–9.2	
FGB	3.9	4.0	3.95 ± 0.064	3.9–4.0	
FL	24.2	22.6	23.43 ± 1.115	22.2–22.6	21.9
FW	12.7	12.8	12.75 ± 0.127	12.7–12.8	10.5
TL	21.9	21.5	21.7 ± 0.219	21.5–21.9	20.2
TW	9.0	9.3	9.15 ± 0.269	9–9.3	7.7
FOL	20.3	22.5	21.41 ± 1.58	20.3–22.5	20.4
TFOL	27.5	29.3	28.39 ± 1.24	27.5–29.3	27.0
T1L	4.8	4.3	4.59 ± 0.349	4.3–4.8	4.2
T2L	5.4	4.5	4.95 ± 0.603	4.5–5.4	4.9
T3L	8.4	7.3	7.84 ± 0.775	7.3–8.4	7.3
T4L	9.8	11.4	10.64 ± 1.13	9.8–11.4	10.4
T5L	9.0	8.5	8.73 ± 0.356	8.5–9	8.2
td1	1.6	1.3	1.45 ± 0.163	1.3–1.6	1.7
td2	1.7	1.5	1.56 ± 0.156	1.5–1.7	1.9
td3	1.5	1.4	1.46 ± 0.094	1.4–1.5	1.9
td4	1.5	1.3	1.4 ± 0.108	1.3–1.5	1.7
td5	1.3	1.3	1.28 ± 0.007	1.3–1.3	1.5
tw1	0.7	0.8	0.725 ± 0.045	0.7–0.8	0.5
tw2	0.8	0.8	0.802 ± 0.064	0.8–0.8	0.6
tw3	0.7	0.7	0.708 ± 0.045	0.7–0.7	0.7
tw4	0.9	0.6	0.767 ± 0.174	0.6–0.9	0.6
tw5	0.7	0.7	0.688 ± 0.002	0.7–0.7	0.6
IMT	4.2	4.0	4.12 ± 0.179	4–4.2	3.3
FFTF	5.8	8.5	7.13 ± 1.93	5.8–8.5	7.5
TFTF	5.4	7.7	6.54 ± 1.61	5.4–7.7	6.9
MTTF	14.6	15.2	14.86 ± 0.41	14.6–15.2	13.7
MTFF	15.0	14.8	14.91 ± 0.15	14.8–15.0	14.1

Forelimbs (FLL 9.6 mm) strong, shorter than hand (HAL 11.4 mm); third finger thin, long (TFL 4.8 mm), rounded in the tip; no dermal fringe on finger III; webbing absent (Fig. 8F); relative length of fingers, shortest to longest: I < II < IV < III; tips of fingers enlarged with weakly developed disc and third finger disc without dorso–terminal groove (Fig. 8H) subarticular tubercles moderately developed, one on fingers I and II, two on fingers III and IV; thenar tubercle oval and palmar tubercle distinct (Fig. 8F).

Hind limbs moderately long; tibia length (TL 21.6 mm) more than two times as long as wide (TW nine mm), shorter than femur length (FL 21.9 mm) and sub-equal to the distance from the base of the internal metatarsal tubercle to the tip of toe IV (FOL 20.3 mm); heels do not touch when tibia folded perpendicular to median plane of body; toes thin and long; toe IV long (T4L 9.8 mm) and less than the distance from the base of the tarsus to the tip of toe IV (TFOL 27.5 mm); relative length of toes: I < II < III < V < IV (Fig. 8G); tips of toes with moderately developed discs; and fourth toe disc with dorso–terminal groove, cover notched distally (Figs. 8G and 8I), webbing moderately developed (I 0–1 II 0–2 III 0–0 IV 3–0 V) (MTTF 14.6 mm; MTFF 15.1 mm; TFTF 5.4 mm; FFTF 5.8 mm) (Fig. 8J); dermal fringe along toe V distinct, touching on the base of length of inner metatarsal tubercle (IMT) on toe I; subarticular tubercles present, moderately large, pear shaped, numbering one on toes I and II, two on toes III and V, and three on toe IV; inner metatarsal tubercle elongate and prominent (IMT 4.3 mm), shorter than toe I (T1L 4.8 mm); outer metatarsal tubercle, supernumerary tubercles and tarsal tubercles absent (Fig. 8G).

Skin corrugated on dorsal and lateral surfaces of body with glandular folds running parallel from snout to vent; dorsal surface of forelimbs and hindlimbs with short longitudinal glandular folds; upper eyelid with four rows of glandular folds running parallel to the eyelid; short longitudinal glandular folds arranged in three arching series between eye and shoulder, four similar folds arranged on dorsolateral sides of trunk from level of shoulder to groin; snout with ‘Y’ shaped fold from middle of upper lip, its arms bifurcating between nares and running towards orbits; supratympanic fold and subocular fold distinct; prominent horizontal fold connecting the upper eyelids at the anterior level; throat with dense glandular longitudinal folds; rest of venter with reticulated glandular wrinkles, without any prominent folds; belly smooth; thigh ventrally smooth (Fig. 8D); dorsally tarsus, heel, and knee with fine spinules (Fig. 8C); femoral gland present near vent, oval, moderately large (femoral gland length (FGL) 9.2 mm; femoral gland breadth (FGB) 3.9 mm).

### Color of holotype in life

Iris golden yellow in its upper part, pupil rhomboidal, black in colour (Figs. 8A and 8B). The sides of the head are light–coloured, and the region between the subocular gland and the tympanic region is cream–coloured. A black horizontal band connects the upper eyelids. Dorsum brown uniformly from the base of the orbits to the vent, merging into light brown to orange colouration on the sides of the body. Forearm barred with small, black and larger dark brown bands; hind limbs barred with small light brick red and larger dark brown bands. The sides of the body are brownish. Nuptial pads on the base of the first fingers are cream–coloured. The throat is translucent with fine black spots on the glandular skin. The chest, belly, and anterior part of the thighs are translucent. The posterior part of the thigh with a raised pale white femoral gland in males.

### Color of holotype in ethanol 70%

Dorsal and lateral parts of body mottled with light brown and cream colour, with a pair of prominent dorsolateral lines from posterior orbit to above vent; faint horizontal band between eyes; femoral glands are light brown to creamish white with granules; dorsal parts of limbs, forelimbs, femur, tibia and foot up to tip of fingers and toes barred; throat and chest brown with granular folds; belly and thighs white with two V–shaped lines starting from the base of axilla and reaches in the middle of ventral chest (Figs. 8C and 8D).

### Secondary sexual characters

Males have a nuptial pad that covers the base of the dorsal surface of the first finger. A pair of femoral glands is present in males, which are more than 2 times longer (FGL 8.7–9.2 mm) than wide (FGB 3.9–4 mm). It is present on the ventral side of the thigh, which is clearly visible both in life and preservative (Fig. 8D). Females have eggs that are pigmented.

### Variation

Morphological data of paratypes and variations are given in Table 4. Male paratype has an SVL of 48.1 mm, the female paratype an SVL of 43.3 mm; in all the external morphological features, they are similar to the holotype.

### Natural History and Distribution

*Nyctibatrachus kali* sp. nov. has been observed from the small river networks in Kali, as shown in Fig. 1. Adults were observed from streams at Castle Rock village, Dandeli Taluk, Uttara Kannada district, Karnataka state, India (15.400894°N, 74.323950°E, 578 m asl).

### Conservation

The localities where *Nyctibatrachus kali* sp. nov. occurs are inside protected areas of Castlerock in Karnataka. Castlerock was once a mining area for Manganese minerals, but mining activities are completely banned now, and the area is now a part of the Kali Tiger Reserve. However, the type locality of *Nyctibatrachus kali* sp. nov. is very close to the Castlerock Railway line (49 m) and to Dudhsagar waterfalls (10 km), a popular tourist area. Besides *Nyctibatrachus kali* sp. nov., *Nyctibatrachus petraeus* (Das & Kunte, 2005), and *Raorchestes bombayensis* (Annandale, 1919) are two other frogs discovered from this region. Overall, there are 30 amphibians known from the Castlerock region, suggesting a good diversity of amphibians. Due to the proposed railway line doubling, transmission lines and road expansion in this region (Punjabi et al., 2020), along with continuous influx of tourists, the habitats of *Nyctibatrachus kali* sp. nov., as well as other frogs, are under threat and require continuous monitoring of the stream habitats.

Based on our field observations, this species is locally abundant in the surroundings of the type locality. Its habitats are torrents and streams of the tropical evergreen and moist deciduous forests of the Western Ghats. Based on our current understanding and distribution, it should be classified as a “Data Deficient” (DD) species according to the criteria of the IUCN Red List (IUCN SSC Amphibian Specialist Group, 2023).

## Discussion

Species in the genus *Nyctibatrachus* are morphologically cryptic and show remarkable similarity in morphology (Kumar et al., 2022). It is quite evident that *Nyctibatrachus kali* sp. nov. is similar to *N. kumbara* and *N. tunga* morphologically but differs genetically and acoustically. Thus, integrative taxonomic studies are essential to delimit species. The phylogenetic analyses showed that *Nyctibatrachus kali* sp. nov. is a member of the *N. sanctipalustris* group (Table S5). The members of this group show allopatric distribution and are geographically distant from *Nyctibatrachus kali* sp. nov. (minimum: 62.7 km with *N*. *kumbara* and 536 km with *N. vrijeuni*). However, Castlerock is on the border of the Goa–Karnataka Western Ghats region. There is only scarce data of the *Nyctibatrachus* species from the Goa region. A fine–scale sampling of streams from Goa may uncover species that are geographically and genetically closer to *Nyctibatrachus kali* sp. nov. It can provide a better understanding of the phylogenetic position of *Nyctibatrachus kali* sp. nov.

Anuran amphibians show specifically distinct advertisement calls (Köhler et al., 2017). These calls help in species discovery and have evolutionary implications (Vázquez-Hernández et al., 2024; Gingras et al., 2013). Our study observed two distinct advertisement calls in *Nyctibatrachus kali* sp. nov. and its congeners for the first time. In single–note and multiple–note calls, we found low–frequency and high–frequency calls (Figs. 5 and 6). This has never been mentioned in any of the *Nyctibatrachus* descriptions. However, in the genus *Indirana*, a similar high and low–frequency call pattern was observed (Modak et al., 2016) in single–note and double–note calls. Single–note calls have high frequency, and double–note calls have one high and one low–frequency note. From our study, we observed that the lower dominant frequency notes showed less variation, but the higher dominant frequency notes showed significant variations between species of *Nyctibatrachus*. These differences in call notes could be important for mate selection. For example, an experimental study by Yue et al. (2017) found that the first note in advertisement calls provides crucial information for auditory recognition by females. The function of the different call types in *Nyctibatrachus kali* sp. nov. and other congeners remains unclear but should be addressed in bioacoustics and ecological studies of these species.

With the addition of *Nyctibatrachus kali* sp. nov., the genus *Nyctibatrachus* currently represents 35 species. Although the systematics of this genus were revised by Biju et al. (2011), cryptic diversity, point endemism, and poor knowledge of range limits hinder understanding of the actual diversity in the *Nyctibatrachus* genus. At present, 24 out of 35 described *Nyctibatrachus* species have narrow distribution ranges (IUCN SSC Amphibian Specialist Group, 2023), with 75% assigned to the Endangered or Threatened category (IUCN SSC Amphibian Specialist Group, 2023). The same applies to *Nyctibatrachus kali* sp. nov. as well as to its sister species, *N. dattatreyaensis*. While *Nyctibatrachus kali* sp. nov. is known presently only from the type locality, *N. dattatreyaensis* also has a restricted distribution (Extent of occurrence–630.73 km^2^), putting it into the Endangered category (IUCN SSC Amphibian Specialist Group, 2023). Further studies with fine–scale sampling across the Western Ghats River basins could result in a better understanding of the distribution ranges of the 35 *Nyctibatrachus* species. This will be helpful in identifying potential threats and aiding in more effective conservation efforts.

## Conclusions

*Nyctibatrachus kali* sp. nov., a newly discovered frog from the Castlerock region of the Western Ghats, is morphologically cryptic and remarkably similar in appearance to close relatives such as *N. kumbara* and *N. tunga*. However, it is distinguished by genetic and acoustic differences, as evidenced by distinct advertisement calls and phylogenetic analyses that place it within the *N. sanctipalustris* group. These findings highlight how integrative taxonomic approaches-including genetics, morphology, and bioacoustics data-are essential to accurately delimit species within *Nyctibatrachus*, a genus known for cryptic diversity and endemism. Fine-scale sampling of streams in underexplored regions like Goa is likely to reveal additional genetically distinct species and provide greater clarity on the evolutionary relationships within this group.

With the addition of *Nyctibatrachus kali* sp. nov., the genus *Nyctibatrachus* now comprises 35 described species, most of which are range-restricted and face substantial threats from habitat disturbance due to infrastructure projects, tourism, and historical land use. The Castlerock region alone supports significant amphibian diversity, but the continued survival of *Nyctibatrachus kali* sp. nov. and related species is jeopardized by fragmented habitats and limited distributions. As 75% of *Nyctibatrachus* species are listed as Endangered or Threatened, ongoing research, comprehensive surveys, and targeted conservation strategies are imperative for protecting these unique amphibians and better understanding the true extent of their diversity and distribution.

## Supplemental Information

10.7717/peerj.20895/supp-1 Supplemental Information 1 Morphometric measurements Abbreviations of morphometric measurements used in this study

10.7717/peerj.20895/supp-2 Supplemental Information 2 Morphological measurements Abbreviations of the morphometric measurements used in this study

10.7717/peerj.20895/supp-3 Supplemental Information 3 ND1 sequences of *Nyctibatrachus kali* sp. nov

10.7717/peerj.20895/supp-4 Supplemental Information 4 The raw morphometric measurements used in this study

10.7717/peerj.20895/supp-5 Supplemental Information 5 Voucher numbers The voucher numbers of specimens used in this study

10.7717/peerj.20895/supp-6 Supplemental Information 6 Data transformation The data transformation for PCA

10.7717/peerj.20895/supp-7 Supplemental Information 7 Genbank accession numbers The GenBank accession numbers of voucher specimens used in this study

10.7717/peerj.20895/supp-8 Supplemental Information 8 Uncorrected pairwise genetic distance

10.7717/peerj.20895/supp-9 Supplemental Information 9 LDA Data transformation and results

10.7717/peerj.20895/supp-10 Supplemental Information 10 MANOVA Analysis

10.7717/peerj.20895/supp-11 Supplemental Information 11 Tukey’s Honestly Significant Difference (HSD) test

10.7717/peerj.20895/supp-12 Supplemental Information 12 Mann Whitney U test for advertisement call parameters

## Additional Information and Declarations

### Field Study Permissions

The following information was supplied relating to field study approvals (i.e., approving body and any reference numbers): Field experiments were approved by the Karnataka Forest Department (PCCF(WL)/E2/CR–49/2018–19).

### DNA Deposition

The following information was supplied regarding the deposition of DNA sequences: The sequences are available at NCBI: 16S rRNA: PV574459 –PV574460 and ND1: PV561076 –PV561077.

### Data Availability

The following information was supplied regarding data availability: The raw data is available in the Supplemental Files.

### New Species Registration

The following information was supplied regarding the registration of a newly described species: *Nyctibatrachus kali* Species LSID: urn:lsid:zoobank.org:act:996A718B-844E-4C7E-A472-15718B6928D9 Publication LSID: urn:lsid:zoobank.org:pub:75020FA4-D172-4703-B1F9-FFCD1AED5256.
