# Supplementary figures and images for: Hiding in plain sight: description of a new species of Nyctibatrachus (Amphibia, Anura, Nyctibatrachidae) from the central Western Ghats, India
Source: PeerJ. 2026 Mar 27;14:e20895. doi: 10.7717/peerj.20895 (PMC13034866; doi:10.7717/peerj.20895)

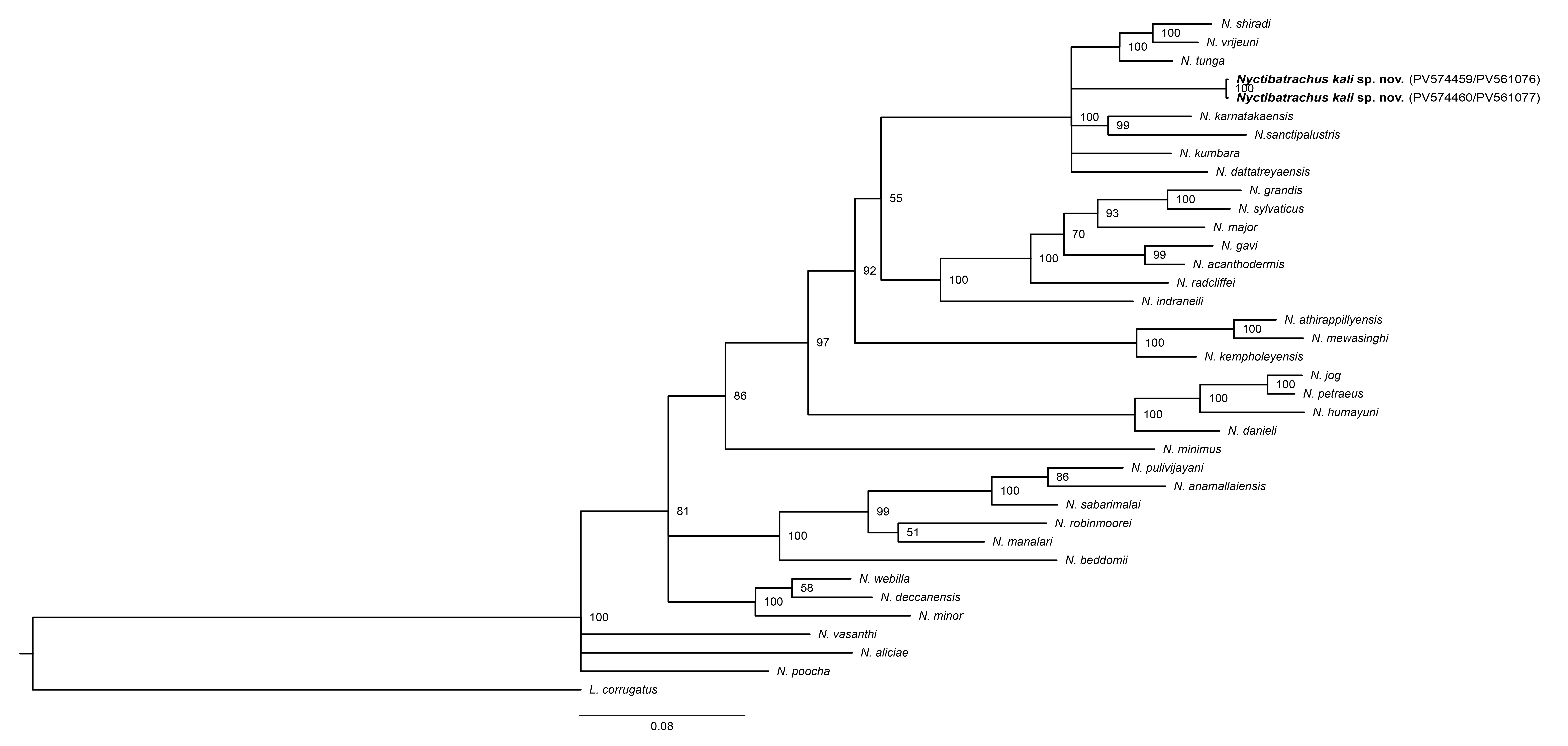

Supplement: Supplemental Information 1 — Abbreviations of morphometric measurements used in this study [file peerj-14-20895-s001.jpg]
