# Supplementary material for: Hiding in plain sight: description of a new species of Nyctibatrachus (Amphibia, Anura, Nyctibatrachidae) from the central Western Ghats, India
Source: PeerJ. 2026 Mar 27;14:e20895. doi: 10.7717/peerj.20895 (PMC13034866; doi:10.7717/peerj.20895)
Supplement: Supplemental Information 2 — Abbreviations of the morphometric measurements used in this study [file peerj-14-20895-s002.docx]

**Morphological measurements**

AG–axilla to groin distance; BW–body width behind shoulders, from right axilla to left axilla; BWG–body width in front of groin; EL–eye length, i.e., the horizontal distance between the bony orbital borders of the eye; EN–eye to nostril distance, i.e., distance between anterior most point of eye and middle of nostril; FD I, II, III and IV–maximum disc width on fingers I, II, III and IV respectively; FFL–first finger length i.e., tip of finger disc to proximal palmar tubercle; FFTF–distance from maximum incurvature of web between fourth and fifth toe to tip of fourth toe; FGB–femoral gland breadth; FGL–femoral gland length; FL–femur length, Femur-tibia articulation (knee) to cloaca; FLL– forelimb length, measured from the elbow to the base of the outer palmar tubercle; FOL–foot length, measured from the base of the inner metatarsal tubercle to the tip of the fourth toe; FrFL–fourth finger length, measured from base of proximal sub–articular tubercle to fingertip; FW–femur width, Maximum width across thigh (femur); fw I, II, III and IV–width of finger I, II, III and IV respectively, measured at the base of the disc; HAL– hand length, measured from the base of the outer palmar tubercle to the tip of the third finger; HD–head depth, measured as a vertical profile at region behind eyes to chest; HL–head length, from the rear of the mandible to the tip of the snout; HW–head width, at the angle of the jaws; IBE–distance between posterior corner of eyes, i.e., the shortest distance between the posterior–most orbital borders of the eyes; IFE–distance between anterior corner of eyes, i.e., the shortest distance between the anterior orbital borders of the eyes; IMT–length of inner metatarsal tubercle; IN–internarial distance, i.e., least distance between the inner margins of nares; IUE–inter upper eyelid width, i.e., the shortest distance between the upper eyelids; MBE–distance from the rear of the mandible to the posterior–most orbital border; MFE–distance from the rear of the mandible to the anterior–most orbital border; MN–distance from the rear of the mandible to the center of the nostril; MTFF–distance from distal edge of metatarsal tubercle to maximum incurvature of web between fourth and fifth toe; MTTF–distance from distal edge of metatarsal tubercle to maximum incurvature of web between third and fourth toe; NS–nostril to snout distance, i.e., distance between middle of nostril and tip of snout; SFL–second finger length; SL–snout length, measured from the tip of the snout to the anterior–most orbital border; SVL–snout to vent length; T1L–toe one length (tip of disc to proximal sub articular tubercle); T2L–toe two length; T3L–toe three length; T4L–toe four length; T5L–toe five length; td I, II, III, IV and V–maximum disc width on toes I, II, III, IV and V respectively; TFL–third finger length; TFOL–distance from the heel to the tip of the fourth toe; TFTF–distance from maximum incurvature of web between third and fourth toe to tip of fourth toe; TL–tibia length; tw I, II, III, IV and V–width of toes I, II, III, IV and V respectively, measured at the base of disc; UEW–maximum upper eyelid width.
